# Integrative profiling of diverse post-translational modifications for prognostic stratification and personalized therapy in papillary thyroid cancer

**DOI:** 10.3389/fendo.2026.1875494

**Published:** 2026-07-27

**Authors:** Kunyi Wang, Fang Li, Yi Zhou, Daqi Zhang, Yantao Fu, Shijie Li, Le Zhou, Qian Ao, Yanqing Lv, Peiyao Wang, Hui Sun, Nan Liang

**Affiliations:** China-Japan Union Hospital of Jilin University, Department of Thyroid Surgery, Jilin Provincial Key Laboratory of Thyroid Disease, Changchun, China

**Keywords:** papillary thyroid cancer, post-translational modification, prognosis, therapy, tumor immune microenvironment

## Abstract

Post-translational modifications (PTMs) are pivotal in tumor biology, yet their role in papillary thyroid cancer (PTC) remains unclear. We integrated bulk and single-cell transcriptomes with clinical data from The Cancer Genome Atlas (TCGA) and Gene Expression Omnibus (GEO) databases to analyze 17 PTMs and construct a prognostic model using 12 machine learning algorithms for predicting the thyroid cancer-free interval (TCFi). Enrichment analysis, single-cell analysis, and immune-related analysis were performed to elucidate the biological role of PTMs. Therapeutic responses of PTC patients were predicted based on the model. We validated the expression of model genes and identified the key signature associated with the malignant phenotypes of PTC. We filtered out 12 genes to construct a post-translational modification index (PTMI) and identified three molecular clusters of PTC. Shapley additive explanations (SHAP) and nomogram models confirmed the predictive efficacy of PTMI. Integrated analyses revealed significant associations between PTMI and immune features. High-PTMI patients showed sensitivity to FDA-approved drugs and chemotherapeutics but resistance to radioactive iodine therapy. Notably, we identified TYMS as a key functional PTMI signature. The PTMI proposed in this study holds strong potential as a prognostic biomarker and therapeutic predictor, offering valuable insights for personalized management of PTC patients.

## Introduction

As the most prevalent endocrine malignancy, more than 821, 000 new cases of thyroid cancer (THCA) were estimated to occur in 2022. With over 466, 000 cases, China alone accounts for over half of the incidence burden worldwide (1). PTC is the most common histology, while mortality due to PTC continues to increase, especially for major diameter and distant metastasis (2). Although most PTCs present an indolent clinical course, several patients already at the advanced stage at diagnosis, even experience recurrence and locoregional or distant metastasis after initial treatment (3). It is associated with significantly poorer outcomes and demonstrably presents additional challenges in the management of both primary and recurrent tumors. Therefore, a reliable stratification signature is essential for guiding individualized treatment approaches to improve the prognosis.

PTMs introduce structural changes in existing proteins to participate in multiple biological processes (4). From an immunological perspective, crotonylation influences the reprogramming of the tumor immune microenvironment (TIME) by regulating immunogenic cytosolic RNA and DNA generation (5), phosphorylation and glycosylation modulate antigen presentation and recognition (6), and mTOR malonylation is essential for the impairment of angiogenesis (7). Additionally, palmitoylation of cyclic guanosine monophosphate‐adenosine monophosphate synthase (cGAS) inhibits the innate immune response that serves as a newly identified PTM (8). In the genetic context, acetylation affects gene transcription and internucleosome interactions (9), and histone butyrylation is associated with active gene regulatory elements and gene expression levels (10).Furthermore, PTMs play a pivotal role in regulating cellular metabolism by dynamically modulating protein activity, stability, and interactions. Ubiquitination is central to lipid metabolism through controlling protein and lipid turnover (11). Nitration of hsp90 functions as a metabolic switch that promotes cell proliferation (12). Moreover, some findings reveal the regulatory role of succinylation in glutaminolysis and tumor growth (13). Meanwhile, crosstalk among diverse PTMs underscores their integrated regulation of cellular processes, which contribute significantly to the dynamic transformation and progression of tumors. Notably, crotonylation and acetylation share common regulatory enzymes, and mitochondrial protein lactylation has been shown to impact oxidative phosphorylation (14). PTMs contribute to protein molecular diversity and dynamic decoration, which is closely associated with tumor initiation and progression (15). Nevertheless, the comprehensive association between PTMs and PTC prognosis and therapy remains unclear.

The objective of this study was to integrate diverse PTMs for prognostic stratification and personalized therapy in PTC. Initially, we analyzed the expression patterns and clinical significance of PTM-related genes in PTC. Subsequently, we developed a PTMI to accurately predict the thyroid cancer-free interval (TCFi) and comprehensively elucidate the relationship between model genes, PTMI, and the carcinogenic mechanisms of PTC. Furthermore, we have investigated the intricate interplay among model genes, PTMI, and TIME. Moreover, we explored the predictive value of PTMI in therapeutic response to immunotherapy and targeted therapy in PTC. Ultimately, we have examined and validated the diagnostic and prognostic implications of hub genes associated with PTMI in PTC.

## Materials and methods

### Data acquisition and preprocessing

Transcriptome profiles and corresponding clinicopathological information of 572 THCA samples were obtained from TCGA (https://portal.gdc.cancer.gov) (16). After excluding cases with incomplete prognostic information or a follow-up period shorter than 4 months, 477 PTC and 59 normal thyroid (NT) samples were included in the final analysis.

In the TCGA cohort, 308 PTC patients received RAI therapy (PTC-RAI), while 169 patients did not undergo RAI. Based on the definition of RAI-refractory outlined in the American Thyroid Association (ATA) guidelines, RAI-refractory disease requires radioiodine whole body scans and serial thyroglobulin, which are not available in TCGA. So we used the presence or absence of TCFi events following RAI therapy as a proxy to stratify PTC-RAI patients into a RAI-sensitive group (RS, n = 268) and a RAI-refractory group (RR, n = 40).

For external validation, expression matrices and platform annotation files for GSE27155, GSE5364, and GSE58545 (platform: GPL96), comprising 113 PTC and 53 NT samples, as well as GSE29265, GSE33630, and GSE60542 (platform: GPL570), including 96 PTC patients and 95 NT samples, were downloaded from the GEO (https://www.ncbi.nlm.nih.gov/geo/) (17). We analyzed the two platforms separately. For GSE5364 we used only the thyroid samples, and for GSE60542 we left out the lymphnode metastasis samples. Within each platform, we combined the datasets after mapping probes to genes and normalizing between arrays, then corrected the dataset batch effect with removeBatchEffect. The data before and after correction are shown in **Supplementary Figures 15a–d**.

To compile the list of PTM-related genes, we integrated data from 17 distinct types of PTMs from multiple reputable scientific sources, including AmiGO, GeneCards, GSEA, Harmonizome, Reactome, relevant review articles (18–34), and manual curation. All gene entries were de-duplicated, and those with strong correlation coefficients were retained, resulting in a final set of 1, 002 PTM-related genes for downstream analysis.

### Identification of the expression and variation levels of PTM-related genes

Raw transcriptome count data of 477 PTC and 59 NT samples were processed for downstream analysis after excluding genes with low expression (average count < 100). Differentially expressed genes (DEGs) between PTC and NT samples were identified using the package ‘DESeq2’. (*P* < 0.05, log2|FC|**≥**1.3) To characterize the somatic mutation landscape of the TCGA-PTC cohort, the “maftools” package was employed. The genomic distribution and variation of PTM-related genes were visualized using a circos plot generated with the “circlize” R package.

### Construction and validation of the PTM-related gene signature

A total of 477 PTC patients were randomly divided into training (n = 287) and testing (n = 190) sets at a ratio of 6:4 using the package ‘caret’ (**Table 1**). The concept of the “thyroid cancer-free interval” (TCFi), defined as the period from four months after initial treatment to events including recurrence, distant metastasis, biochemical evidence of disease and death with tumor, was adopted from Li.et al (35). Initially, univariate Cox regression was utilized in the training set to evaluate the association between PTM-related genes and TCFi. A liberal *P* value threshold of 0.1 was set as a permissive pre-filter for dimensionality reduction. The univariate screening was performed within the training set alone, the testing set was not used for screening or model fitting, and the final signature was determined by the cross-validated machine-learning procedure. Subsequently, to construct a consensus model with high accuracy and stability, we integrated 12 machine learning algorithms and 183 combinations. The comprehensive algorithms included random survival forest (RSF), obliqueRSF, CoxBoost, xGBoost, generalized boosted regression modeling (GBM), Ridge regression, partial least squares regression for cox models (plsRcox), elastic network (Enet), survival support vector machine (survival-SVM), supervised principal components (SuperPC), stepwise Cox (StepCox), and Lasso regression. Model construction was performed using ten-fold cross-validation in the training set, and performance was evaluated in the testing set. Ultimately, the model achieving the highest average Harrell’s concordance index (C-index) was considered optimal. But we note that the testing cohort may have contributed to model selection. Among the 183 algorithm combinations, the one achieving the highest mean Harrell’s C-index across the training and testing cohorts (RSF + obliqueRSF) was selected as the final model. The ‘corrr’ package was employed to display the correlation between model genes.

**Table 1:** Comparison of demographic and clinical characteristics between the training set (N = 287) and testing set (N = 190) of papillary thyroid cancer patients.

Characteristics	Testing set (N = 190)	Training set (N = 287)	P-value
Age			
<55	133 (70.0%)	191 (66.6%)	0.49
>=55	57 (30.0%)	96 (33.4%)	
Sex assigned at birth			
Female	141 (74.2%)	204 (71.1%)	0.52
Male	49 (25.8%)	83 (28.9%)	
T category			
T1	57 (30.0%)	79 (27.5%)	0.86
T2	64 (33.7%)	93 (32.4%)	
T3	61 (32.1%)	103 (35.9%)	
T4	8 (4.2%)	12 (4.2%)	
N category			
N0	107 (56.3%)	159 (55.4%)	0.92
N1	83 (43.7%)	128 (44.6%)	
M category			
M0	185 (97.4%)	283 (98.6%)	0.53
M1	5 (2.6%)	4 (1.4%)	
AJCC 8th TNM			
Stage I	153 (80.5%)	228 (79.4%)	0.95
Stage II	30 (15.8%)	47 (16.4%)	
Stage III~IV	7 (3.7%)	12 (4.2%)	
Progression events			
No	171 (90.0%)	264 (92.0%)	0.56
Yes	19 (10.0%)	23 (8.0%)	
DSS (years)			
Mean (SD)	3.36 (2.90)	3.12 (2.42)	0.34
Median [Min, Max]	2.50 [0.403, 14.9]	2.55 [0.334, 14.1]	

Samples were stratified into distinct PTMI subgroups based on the median PTMI. Kaplan–Meier (KM) curve with log-rank test and receiver operating characteristic (ROC) curve analysis were conducted using the ‘survminer’ and ‘timeROC’ packages. The distribution of PTMI subgroups across the training set, the testing set, and the entire cohort was visualized using the ‘Rtsne’ package. The associations between PTMI and clinical characteristics were analyzed and illustrated using the ‘pheatmap’ package.

### Unsupervised clustering of PCD-related model genes

According to the model genes, we used the “ConsensusClusterPlus” package to complete consensus clustering to identify distinct PTM-related phenotypes in PTC. The clustering was conducted with the following parameters: maxK = 9, pItem = 0.7, clusterAlg = km, distance = euclidean. The TCFi of PTC patients across identified clusters was compared using KM analysis.

### Establishment of SHAP and nomogram integrating the PTMI

In the entire cohort, both univariate and multivariate Cox regression analyses were applied to assess the independent prognostic value of the PTMI. To further validate its predictive importance, SHAP values were evaluated to corroborate the significance of PTMI using the ‘shapviz’ package. Moreover, a nomogram integrating PTMI and clinical features was developed to estimate the probability of 1-, 3-, and 5-year TCFi using the ‘rms’ package. The predictive performance of the nomogram was compared with established risk stratification systems, including the ATA guidelines, the Metastases, Age, Completeness of Resection, Invasion, Size (MACIS) score, the 8th edition of the American Joint Committee on Cancer (AJCC) TNM staging system, and the European Organization for Research and Treatment of Cancer (EORTC) classification system (36–38). Model performance was evaluated using calibration curves, decision curve analysis (DCA) curves, ROC curves, and C-index.

### Functional enrichment analysis

We employed the ‘clusterProfiler’ package to identify significantly enriched biological pathways. We utilized the ‘GSVA’ package to compare the enrichment scores of 25 thyroid-related signatures via single-sample gene set enrichment analysis (ssGSEA). Additionally, gene set variation analysis (GSVA) was performed to evaluate the association between PTMI signatures and 50 canonical hallmarks of cancer (39).

### Single-cell sequencing analysis

Single-cell RNA sequencing data of PTC obtained from the GEO database (GSE250521) were analyzed using the ‘Seurat’ package. To explore the cell-cell interactions among different cell types, we used the ‘CellChat’ package. Besides, the ‘Monocle3’ package was utilized to characterize the dynamic cellular trajectory during PTC progression.

### Tumor microenvironment analysis

We used the ‘estimate’ package to estimate the tumor microenvironment (TME) scores, including immune scores, stromal scores, and tumor purity. To comprehensively characterize immune infiltration, we integrated TIMER, XCELL, QUANTISEQ, MCPCOUNTER, EPIC, and CIBERSORT algorithms to calculate the relative abundance of infiltrating immune cells. Furthermore, to assess antitumor immune response, we obtained the scores for the seven-step cancer-immunity cycle from the Tracking Tumor Immunophenotype (TIP, http://biocc.hrbmu.edu.cn/TIP) (40).

### Assessment of response to multiple therapeutics

For immunotherapy, we dissected the association between PTMI and both the Tumor Immune Dysfunction and Exclusion (TIDE, http://tide.dfci.harvard.edu) (41) score and the immunophenoscore (IPS) obtained from the Cancer Immunome Atlas (TCIA, https://tcia.at/home) (42). For molecular targeted therapy and chemotherapy, we performed drug sensitivity analysis using the ‘oncoPredict’ package, integrating data from the Genomics of Drug Sensitivity in Cancer (GDSC) and the Cancer Therapeutics Response Portal (CTRP) databases.

### Cell culture

The N-thy, TPC-1, BCPAP, FTC, KHM, and CAL cell lines were purchased from Procell (Wuhan, China). N-thy, BCPAP, FTC, KHM, and CAL cells were cultured in RPMI-1640 medium (Thermo, Suzhou, China) containing 10% FBS, whereas TPC-1 cells were cultured in DMEM medium (Thermo, Suzhou, China) containing 10% FBS.

### Transfection and western blot

Cells were seeded into 6-well plates with 70˜90% confluence, and TYMS-siRNAs were transfected with Lipo-3000 (GENEWIZ, Tianjin, China) at the final concentrations of 100 nM. The cells were collected for follow-up experiments after 48 h. The sequences of TYMS siRNAs were as follows: siRNA#1 (sense: GCUGACAACCAAACGUGUGUUTT; antisense: AACACACGUUUGGUUGUCA-GCTT), siRNA#2 (sense: CCCUGACGACAGAAGAAUCAUTT; antisense: AUGA-UUCUUCUGUCGUCAGGGTT), and siRNA#3 (sense: CAGGUGACUUUAUACA-CACUUTT; antisense: AAGUGUGUAUAAAGUCACCUGTT).

For protein extraction, PTC cells were lysed with RIPA buffer (Beyotime, Shanghai, China). Denatured protein samples were separated by 8% SDS-PAGE gel and transferred to the PVDF membrane (Cytiva, Shanghai, China). Then, the PVDF membrane was blocked with 5% normal non-fat milk in TBST for 60 minutes at 26 °C to reduce non-specific binding. TYMS antibody (Proteintech, 1:6000, Wuhan, China) and β-actin antibody (1:20000, Proteintech, Wuhan, China) were applied for primary antibody incubation overnight, goat anti-rabbit and goat anti-mouse IgG (H + L) HRP (1:2000, Proteintech, Wuhan, China) were applied for secondary antibodies for 60 min at 26 °C. The ECL (Beyotime, Shanghai, China) was used for imaging. The optical density of the bands was analyzed and calculated using Image Lab software.

### Cell counting kit-8 assay

For the CCK-8 assay, cells were seeded into 96-well plates and transfected as described above. At 0, 24, 48, and 72 h post-transfection, CCK-8 reagent was added to each well, followed by incubation at 37 °C for 2 h. The survival rates were then calculated by detecting the optical density (OD) of each well using a microplate reader at a 450 nm wavelength.

### Colony formation assay

For the colony formation assay, approximately 500 tumor cells were seeded into each well of a 6-well plate and allowed to grow for a period of 10 days. After the incubation period, the cells were fixed with 4% paraformaldehyde for 30 min. Subsequently, the cells were stained with crystal violet at room temperature for 30 min before the calculation. ImageJ software was used to calculate the cell colonies after washed with PBS.

### Wound healing assay

In the wound healing assay, cells were seeded into 6-well plates and cultured in DMEM supplemented with 10% FBS. Then, the confluent cells were scratched with the tip of a 200 µL pipette and washed three times with PBS to remove cell debris. After 24 h of incubation, cell migration into the wound area was observed and photographed under a microscope.

### Statistical analysis

All statistical analyses and graphical visualizations were performed using R software (version 4.4.2) and RStudio. The unpaired t-test or the Mann–Whitney U test was applied for the comparisons between normally and non-normally distributed variables. The Kruskal–Wallis H test was applied for the comparisons among multiple groups. The chi-square test was applied for the analysis among categorical variables. The Spearman correlation analysis was applied for accessing the correlation between variables. A two-tailed ***P*** value < 0.05 was considered statistically significant.

### External validation of PTMI signatures

The protein expression levels of model genes in PTC and NT tissues were verified by immunohistochemical (IHC) staining data retrieved from the Human Protein Atlas (HPA, https://www.proteinatlas.org/) (43). In addition, model gene expression profiles of 16 thyroid cancer cell lines were obtained from the Cancer Cell Line Encyclopedia (CCLE, https://portals.broadinstitute.org/ccle) (44).

## Results

### The workflow of this study

**Figure 1:**
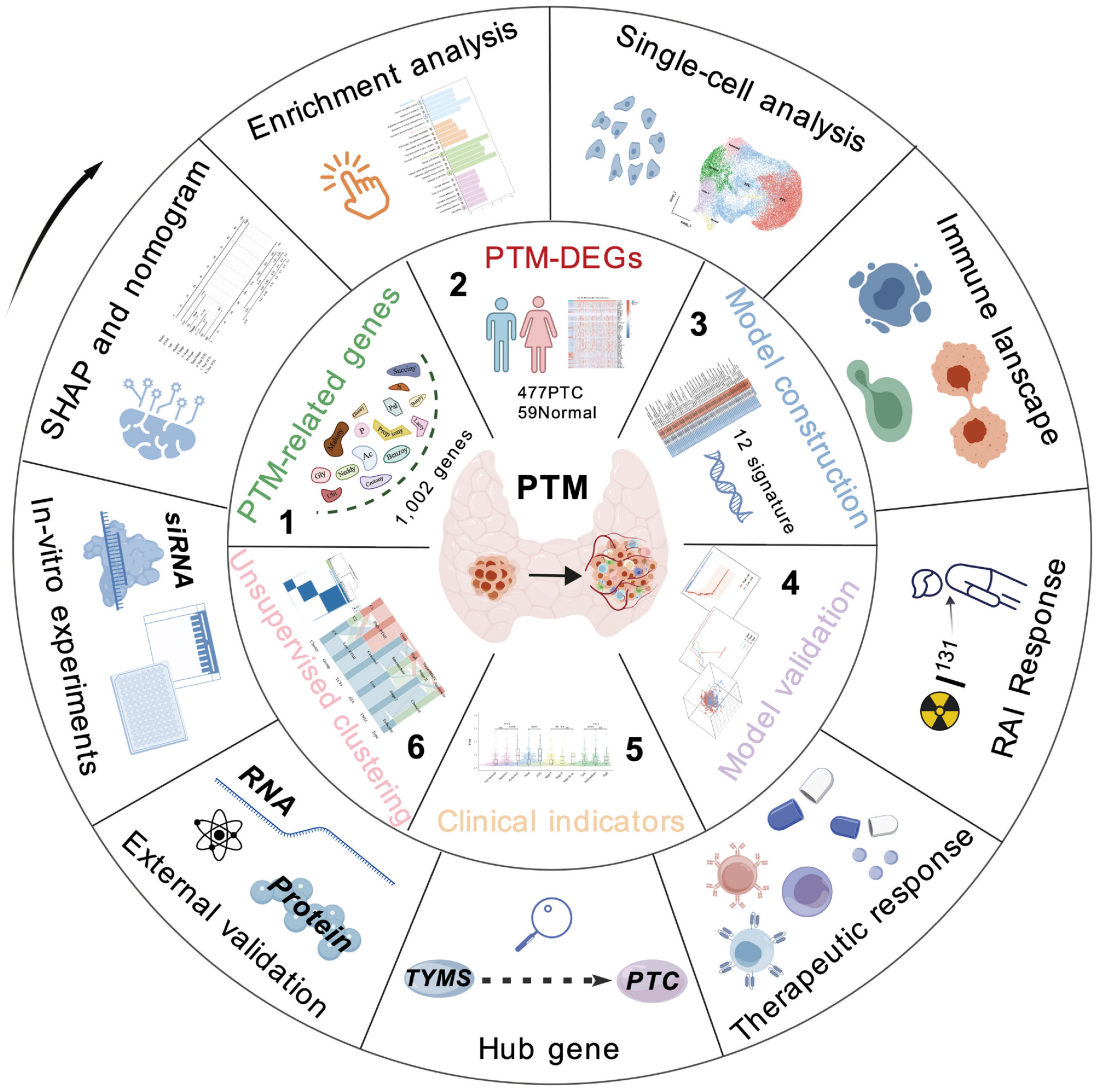
Graphic abstract of this study. Created with BioGDP.com.

We reanalyzed the TCGA dataset to train and validate our comprehensive model from multiple perspectives. For the single-cell RNA transcriptome analysis, we collected data from nine samples, as detailed in GSE250521. Moreover, we gathered external validation data from six GEO datasets: GSE27155, GSE5364, GSE58545, GSE29265, GSE33630, and GSE60542. In total, we examined seventeen PTMs involving a combined set of 1, 002 genes. The study workflow is illustrated in **Figure 1** (45).

### Variant landscape of PTM genes in THCA patients.

**Figure 2:**
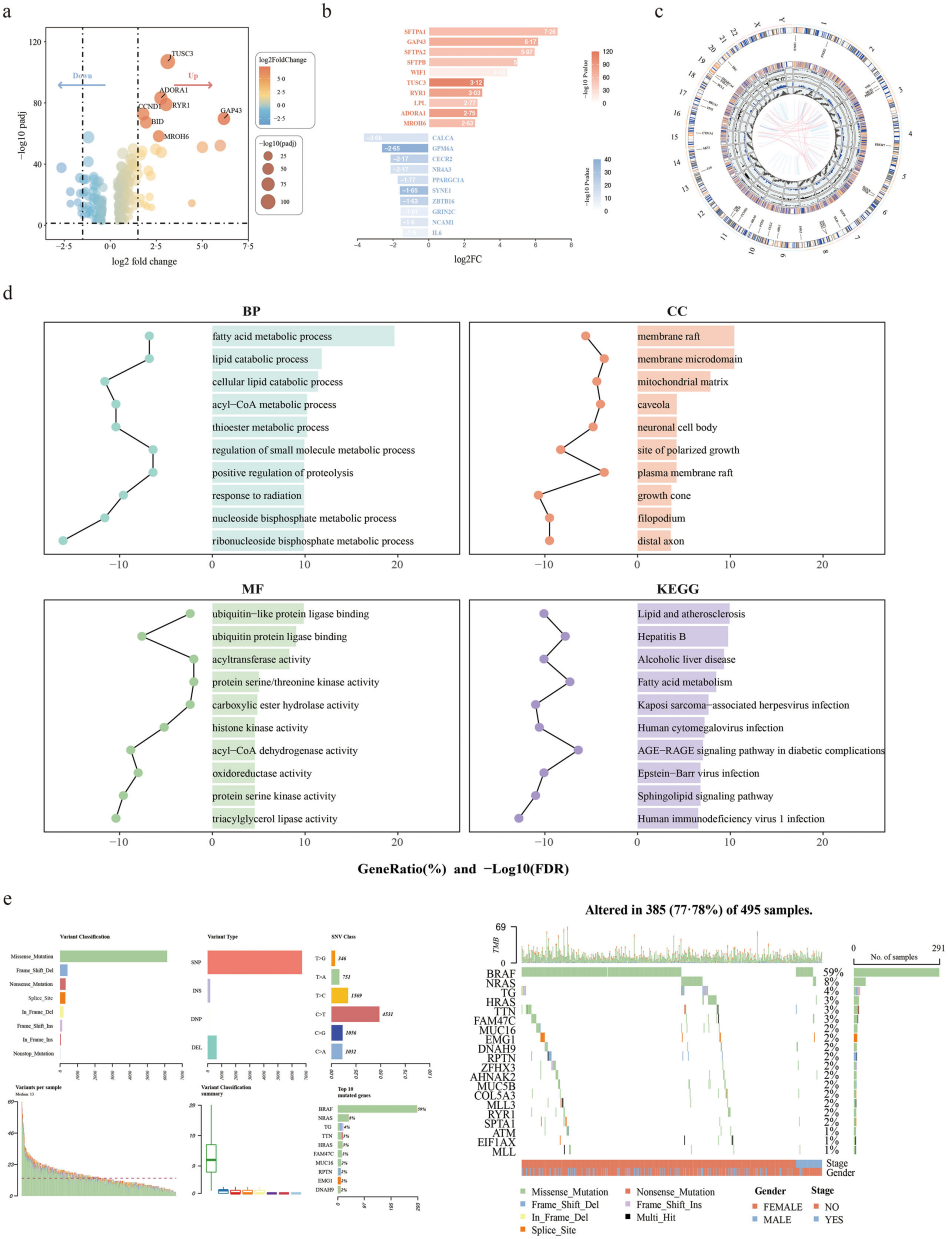
Variant landscape of PTM genes in THCA patients. **(a)** Volcano plot of the 256 PTM-DEGs between PTC and normal thyroid tissues. **(b)** Barplot of top20 PTM-DEGs. **(c)** Circos plot depicting the location, expression, and correlation of PTM-DEGs in the TCGA cohort. **(d)** GO and KEGG enrichment analyses based on the PTM-DEGs. **(e)** An oncoplot of PTM related genes in the TCGA cohort.

In the TCGA-PTC cohort, we identified 256 differentially expressed genes (DEGs) with statistical significance (*P* < 0.05, log2|FC| ≥ 1.3), including 148 upregulated and 108 downregulated genes (**Figure 2a**). A complete list of DEGs is provided in **Supplementary Table 1**. A heatmap displaying the scaled RNA expression levels of the top 50 PTM-DEGs is shown in **Supplementary Figure 1**, while a barplot of the top 20 PTM-DEGs is presented in **Figure 2b**. Additionally, the chromosomal locations, expression patterns, and correlations of PTM-DEGs are illustrated in **Figure 2c**. Moreover, Kyoto Encyclopedia of Genes and Genomes (KEGG) and Gene Ontology (GO) enrichment analysis revealed that these PTM-DEGs are involved in various cancer-related signaling pathways (**Figure 2d**). We further assessed the genomic alterations of PTM-related genes in the TCGA-THCA cohort. Our analysis showed that approximately 77.78% (385/495) of THCA patients harbored mutations. The top 20 gene mutations are displayed, with BRAF exhibiting the highest mutation frequency (19%) and others ranging from 1% to 8% (**Figure 2e**; **Supplementary Figure 2**).

### Construction and validation of a prognostic gene signature via a machine learning-based integrative procedure for PTC patients

**Figure 3:**
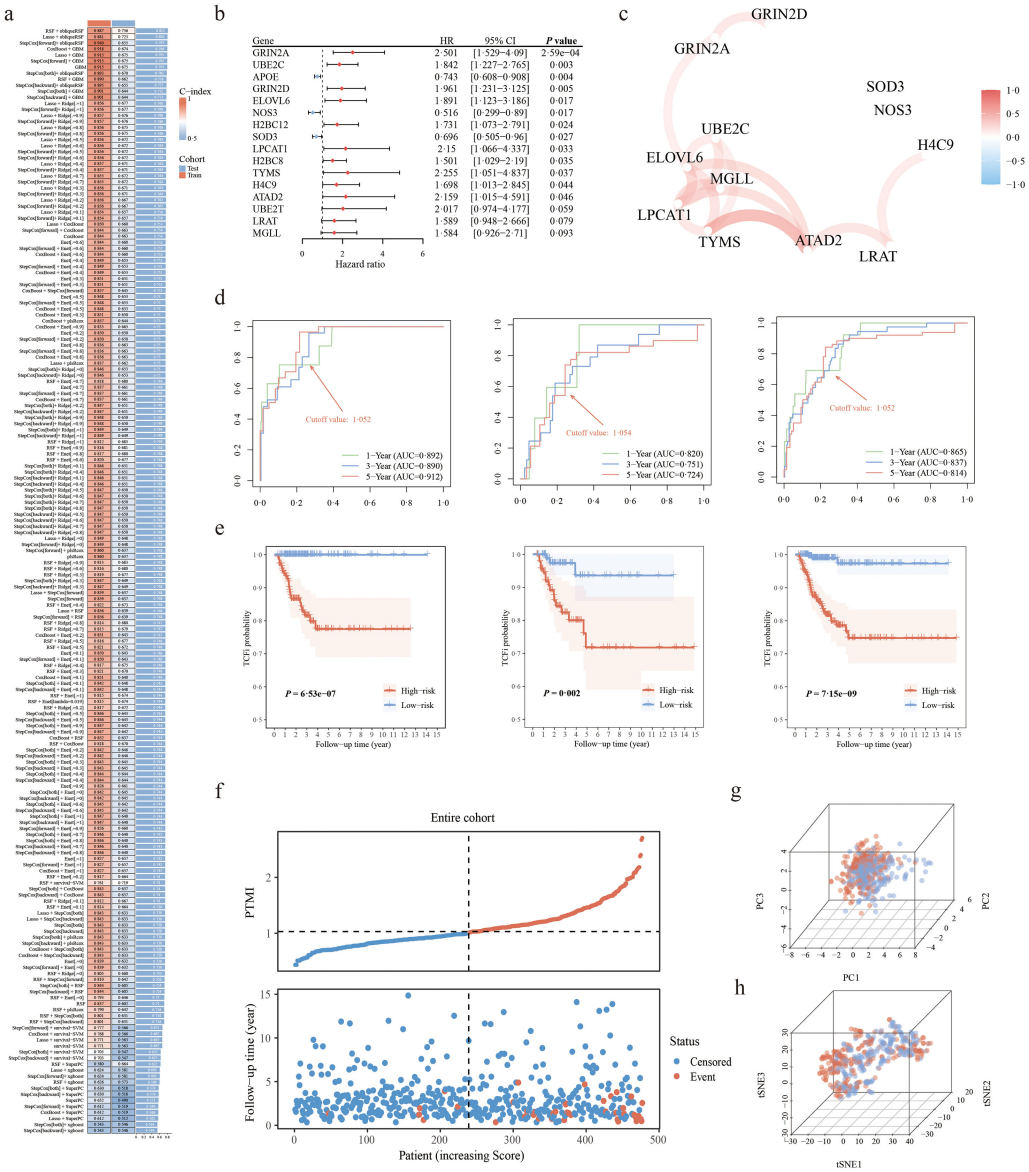
A consensus PTMI was developed and validated via the machine learning based integrative procedure. **(a)** A total of 183 kinds of prediction models via a ten-fold cross-validation framework further calculated the C index of each model. **(b)** Univariate Cox regression analyses of PTM-DEGs associated with TCFi. The top genes with a P value < 0.1 are presented. **(c)**Network of the correlations of the PTM-DEGs. **(d, e)** TCFi ROC curves and KM curves of PTMI subgroups in the training set, testing set, and entire cohort. **(f)** PTMI distribution and prognostic status of each patient. **(g, h)** PCA plot and t-SNE plot show the separation of PTMI subgroups.

To better quantify the PTM level and further demonstrate the predictive value for TCFi, we developed and validated the PTMI based on PTM-DEGs. In the training set, we employed univariate Cox regression analysis preliminarily for the general screening of PTM-DEGs associated with TCFi, and then implemented 183 models integrating 12 distinct machine learning algorithms to determine the optimal approach (**Figures 3a, b**). A 12-gene signature was ultimately constructed using the RSF + obliqueRSF method. Among the selected genes, three were related to nitration, three were related to palmitoylation, two were related to benzoylation, one was related to acetylation, one was related to lactylation, one was related to malonylation, and one was related to ubiquitination. The correlations among model genes were demonstrated in **Figure 3c**; **Supplementary Figure 3**. The PTMI for each patient was the risk predicted by the obliqueRSF model and was used as such in all downstream analyses. The relative importance of each gene in the model was: UBE2C, 0.04; NOS3, -0.87; SOD3, -0.29; LPCAT1, 0.21; ELOVL6, -0.20; H4C9, 0.85; GRIN2A, 0.52; LRAT, 0.53; TYMS, 0.33; MGLL, 0.67; GRIN2D, 0.07; ATAD2, 0.24. In the training set, ROC curves showed that the PTMI achieved AUCs of 0.892, 0.890, and 0.912 for predicting 1-, 3-, and 5-year TCFi, respectively (**Figure 3d**). K-M curves showed that the high-risk group had a significantly shorter TCFi compared to the low-risk group (*P* = 6.53e-07) (**Figure 3e**). Similar predictive performance was observed in both the testing set and the entire cohort. Furthermore, the results of PCA and t-SNE dimensionality reduction analysis confirmed clear separation between the high- and low-risk groups (**Figures 3f–h**; **Supplementary Figure 4**). The above results collectively suggest that the PTMI is a robust and effective tool for predicting TCFi in PTC patients.

### PTMI is strongly associated with clinical characteristics in PTC patients

**Figure 4:**
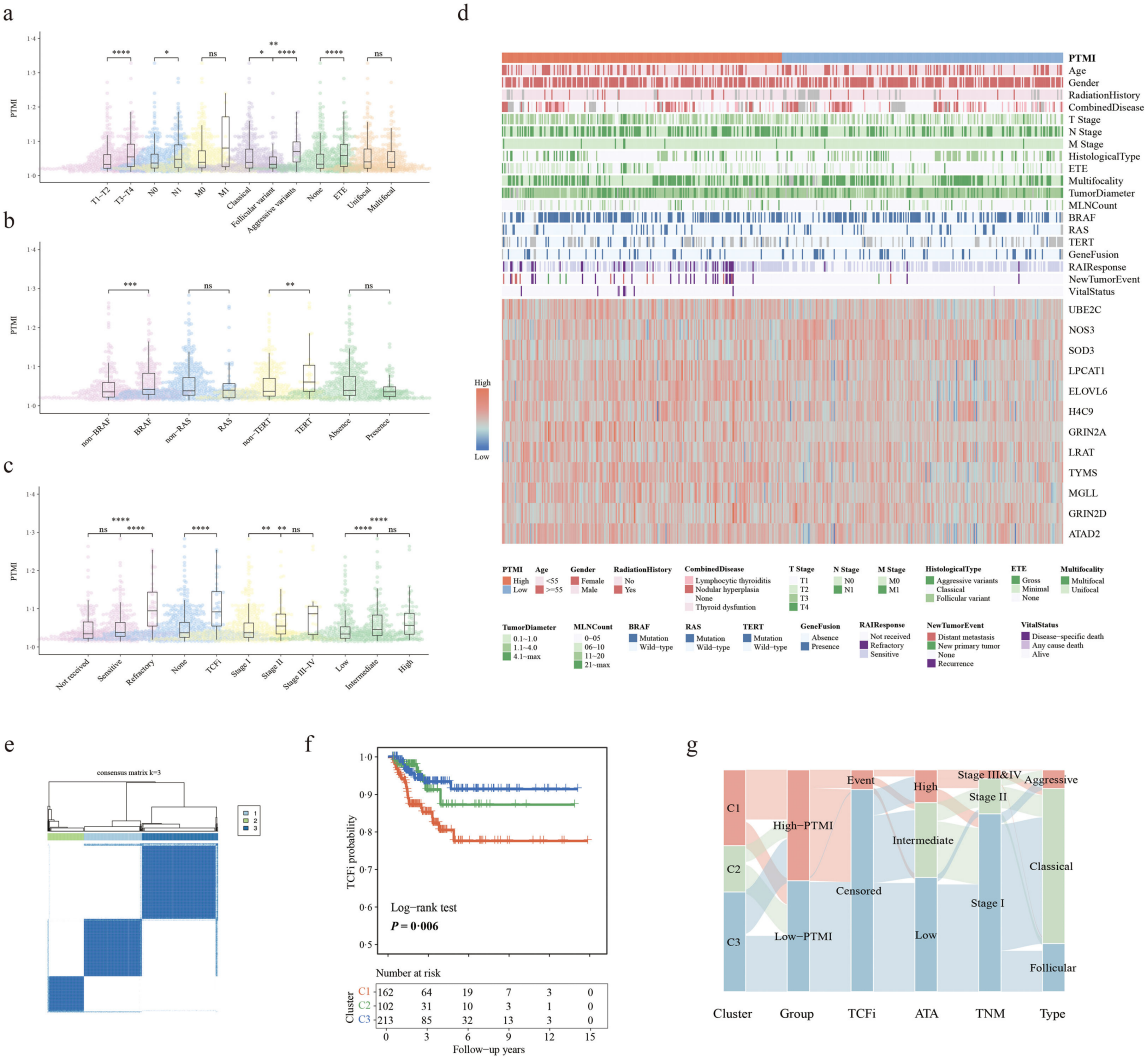
Correlation between the PTMI and clinical indicators. **(a–c)** Barplots of the relationship between PTMI and clinicopathological, molecular, and prognostic features. **(d)** Heatmap of 12 model genes and clinical factors. **(e)** Consensus clustering matrix shows that the 477 PTC patients from the TCGA dataset were classified into three molecular clusters (k = 3) based on the PTM model gene profile. **(f)** The KM curves for TCFi of PTC patients belonging to three different molecular clusters. **(g)** Sankey diagram shows the interrelationship between molecular clusters, PTMI subgroups, survival status, ATA, TNM, and histological type in PTC patients.****Means *P* < 0.0001; ***Means *P* < 0.001;**Means *P* < 0.01; *Means *P* < 0.05; ns Means not significant.

To investigate the association between PTMI and clinical characteristics in PTC patients, we conducted a comparative analysis of clinicopathological, molecular and prognostic features between PTMI subgroups. The high-PTMI group appeared to be more invasive compared to the low-PTMI group, with a higher proportion of patients with advanced stage, aggressive histological variants, and extrathyroidal extension (ETE), as well as molecular alterations primarily involving BRAF, TERT, and model gene mutations, all of which were associated with worse prognosis (more RAI-refractory cases and increased incidence of new tumor events) (**Figures 4a–d**). To further explore previously unidentified subtypes of PTC, we leveraged model genes to stratify the PTC patients into three distinct clusters (**Figure 4e**). Patients in cluster 1 (C1) exhibited the worst prognosis (*P* = 0.006) (**Figure 4f**). Moreover, the Sankey diagram visually illustrated that the majority of patients in C1 were characterized by high PTMI, advanced stage and aggressive histological type. C2 mainly consisted of patients with intermediate-stage disease, while most patients in C3 presented with early-stage disease and low PTMI (**Figure 4g**).

### SHAP values and nomogram model provide additional confirmation of the predictive efficacy of PTMI

**Figure 5:**
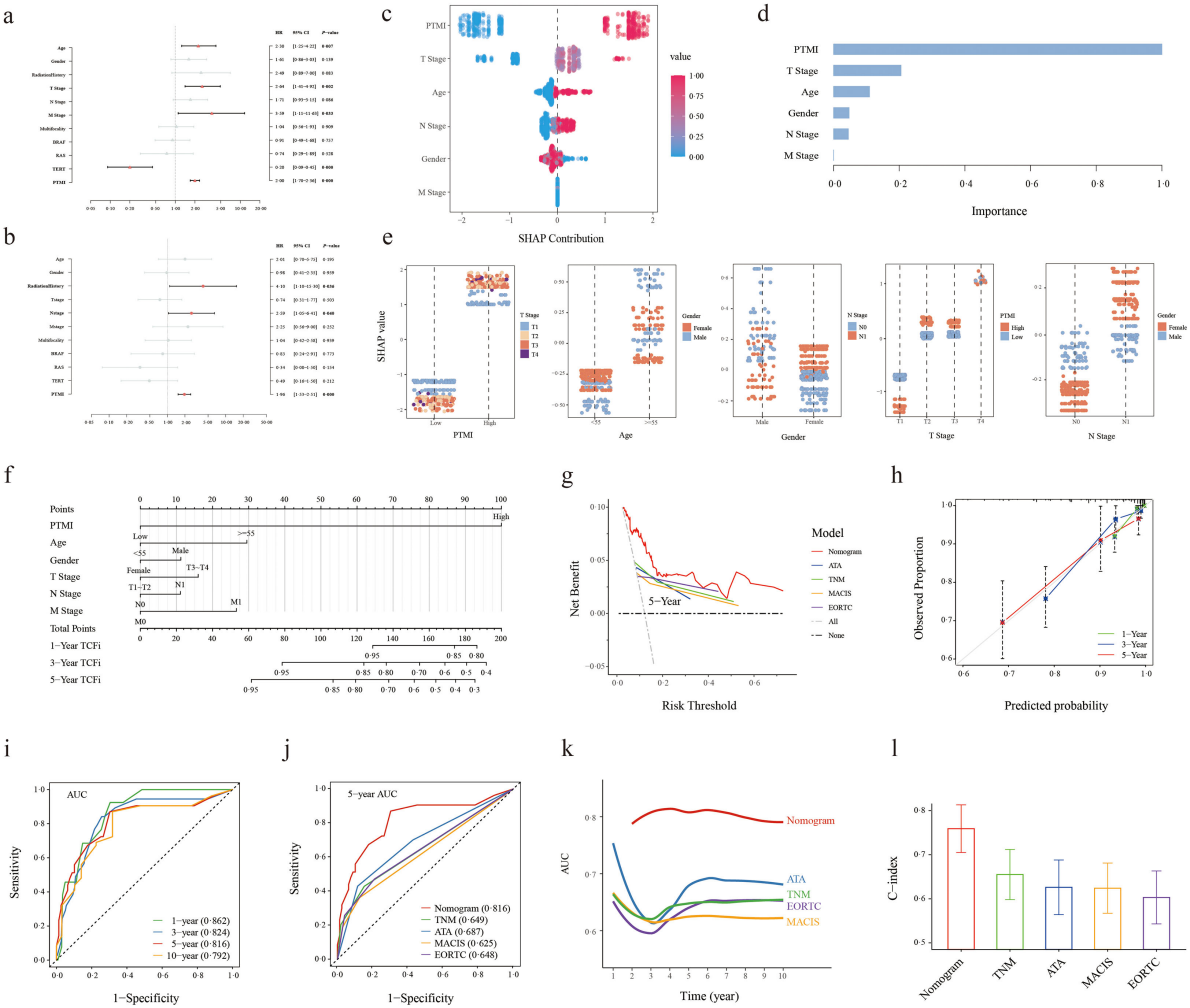
Development and evaluation of SHAP and nomogram values integrating the PTMI in the entire cohort. **(a, b)** Univariate and multivariate Cox regression analyses for validating the independent prognostic value of the PTMI. **(c)** SHAP summary plot. **(d)** SHAP value ranking of the variables in the model. **(e)** Interaction effect diagram of the variables in the model. **(f)** A nomogram to predict the 1-, 3-, and 5-year TCFi. **(g)** DCA curves of the nomogram and four staging systems for predicting the net benefit of the 5-year TCFi. **(h)** Calibration curves of 1-, 3and 5-year TCFi probability for the nomogram. **(i)** Time-dependent ROC curves of the nomogram to predict the 1-, 3-, 5-, and 10-year TCFi. **(j)** ROC curves of the nomogram and four THCA staging systems to predict the 5-year TCFi. **(k)** AUC for the nomogram and four staging systems based on the time-dependent ROC curves. **(l)** C-index of the nomogram and four staging systems.

To assess the independent prognostic value of PTMI, we conducted both univariate and multivariate Cox regression analyses. PTMI was positively and independently associated with TCFi in PTC (HR = 1.96, *P* < 0.001). Age, radiation history, T stage, N stage, M stage, and TERT gene mutation were also significantly related to TCFi (**Figures 5a, b**). Considering sample characteristics and previously established risk stratification systems, we calculated SHAP values integrating age, gender, T stage, N stage, M stage, and PTMI. The results demonstrated that PTMI exhibited superior stratification capability and predictive power (**Figures 5c, d**; **Supplementary Figure 5**). Additionally, PTMI showed a strong interaction with T stage (**Figure 5e**). We then constructed a nomogram model to predict 1-, 3-, and 5-year TCFi of PTC patients based on the above findings (**Figure 5f**). The 5-year DCA curves confirmed that the nomogram provided greater net benefit than other risk stratification systems for TCFi prediction (**Figure 5g**). The calibration curves depicted that the nomogram had excellent agreement between predicted and observed TCFi at 1, 3, and 5 years (**Figure 5h**). ROC curves indicated that the nomogram achieved high AUC values for predicting TCFi at 1, 3, 5, and 10 years (0.862, 0.824, 0.816, and 0.792, respectively) (**Figure 5i**). We further compared the nomogram with the ATA, EORTC, MACIS, and TNM staging systems (**Figures 5j–l**). The nomogram demonstrated superior discriminatory ability, with higher time-dependent AUCs from 1- to 10-year TCFi and a favorable C-index for 5-year TCFi prediction. Overall, SHAP values and the nomogram model provide robust confirmation of the predictive efficacy of PTMI for TCFi in PTC patients.

### Pathway enrichment levels vary among different PTMI patterns of PTC

**Figure 6:**
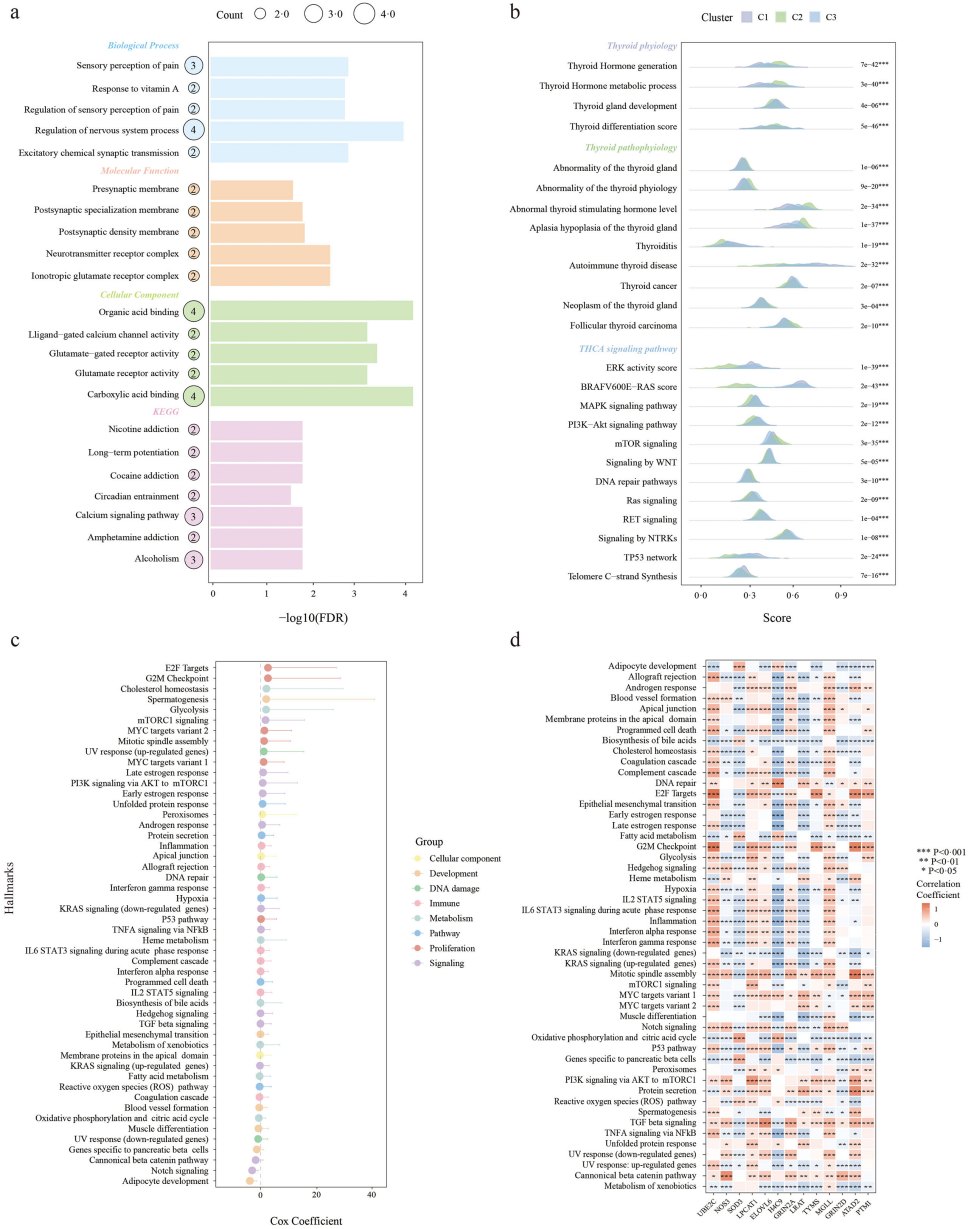
Signaling pathway enrichment analysis of the PTM signatures. **(a)** GO and KEGG enrichment analyses based on the PTMI. **(b)** The ridgeline chart shows the distribution differences in ssGSEA scores of THCA characteristic signatures in three clusters (Kruskal–Wallis H test). **(c)** Univariate Cox regression analysis indicates the risk factor among various hallmarks of cancer. **(d)** The heatmap shows the Pearson correlation between GSVA enrichment analysis scores of 50 hallmarks of cancer and PTMI signatures. ***Means *P* < 0.001; **Means *P* < 0.01; *Means *P* < 0.05.

To elucidate the underlying signaling mechanisms associated with distinct PTMI subgroups, GO and KEGG enrichment analyses were performed based on the model genes (**Figure 6a**). These genes were predominantly enriched in neuroregulatory processes (e.g., regulation of nervous system process), signal transduction pathways (e.g., ligand−gated calcium channel activity), and addiction-related pathways (e.g., nicotine addiction). These findings suggest that PTC cells may aberrantly activate neuronal-immune circuits and calcium/glutamate signaling to promote tumor progression and adaptation. In addition, the enrichment of addiction-associated pathways implies a possible reprogramming of reward-like intracellular signaling that supports tumor cell survival. To further explore the functional heterogeneity, we analyzed the ssGSEA scores of 25 thyroid-related signatures across different clusters (**Figure 6b**). C2 displayed a RAS-like tumor phenotype, characterized by a higher thyroid differentiation score (TDS) and elevated DNA repair activity, but lower oncogenic signaling activation, including reduced ERK pathway signaling. In contrast, C1 showed a BRAF-like profile, with the lowest TDS, and pronounced activation of autoimmune responses, MAPK, and TP53 signaling pathways. C3 presented a mixed phenotype, with co-activation of both PI3K-Akt and Ras signaling pathways. Cox regression analysis identified that E2F targets and G2M checkpoint pathways were strongly associated with poor prognosis, whereas adipocyte development emerged as the most prominent protective factor (**Figure 6c**). Among the 50 cancer hallmarks, PTMI was most positively correlated with programmed cell death, MYC targets, E2F targets, G2M checkpoint, and glycolysis, while it was negatively associated with adipocyte development, muscle differentiation and metabolism of xenobiotics (**Figure 6d**). Together, these results indicate that PTMI may contribute to PTC pathogenesis through regulating immune process and metabolism reprogramming.

### Single-cell analysis reveals a strong association between PTMI and the development and progression of PTC

**Figure 7:**
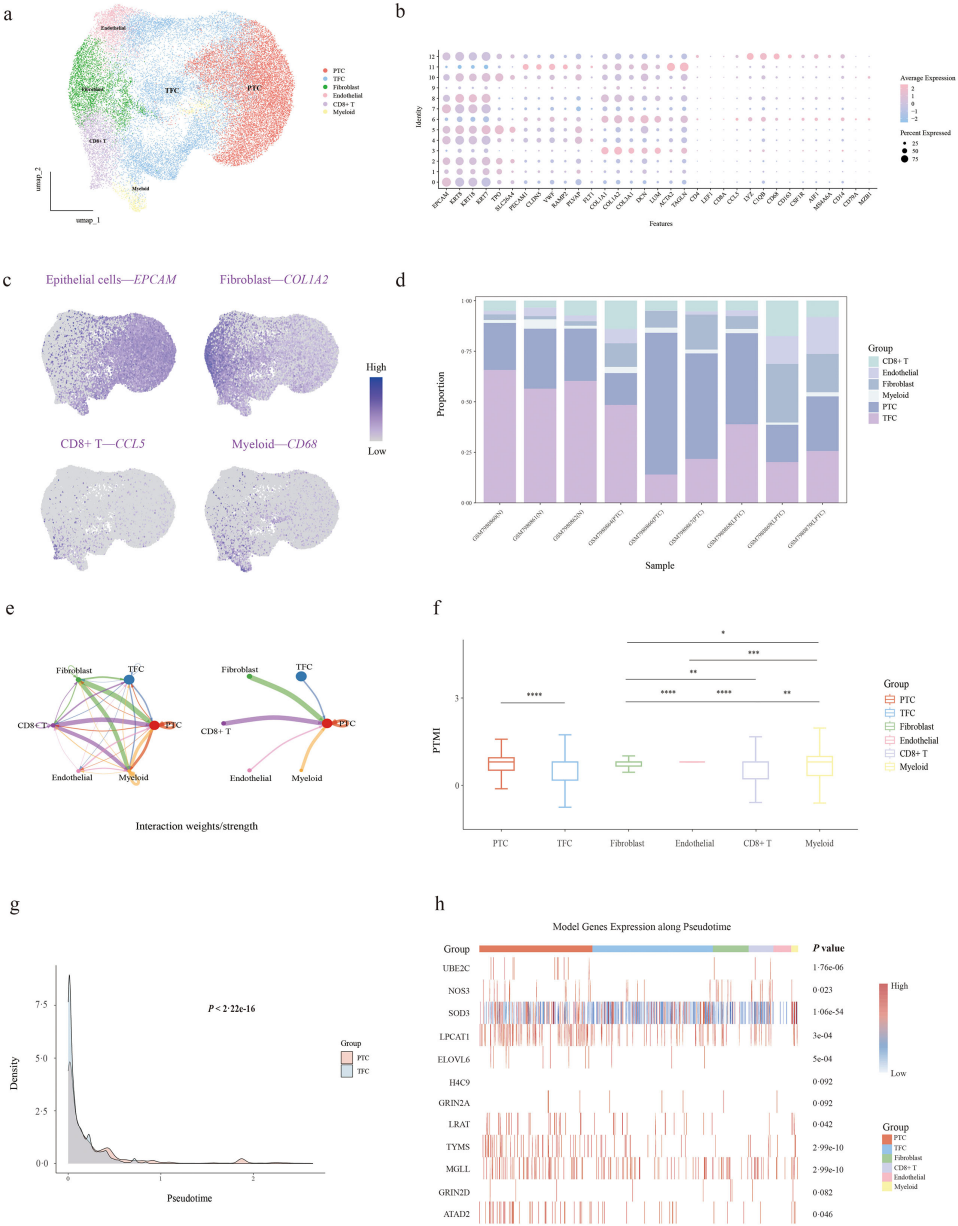
Dissection of tumor microenvironment based on the PTMI signatures. **(a)** UMAP plots for 31916 cells from 9 samples. **(b)** Bubble plot of marker gene expression. **(c)** Feature plots present the typical marker gene expression for major 4 cell types. **(d)** Barplots of the cell proportions in each sample. **(e)** Network of cell-cell interactions among 6 cell types. **(f)** Boxplot of PTMI value in different cell types. **(g)** Density plot of the PTC and TFC along the inferred trajectory. **(h)** Heatmap of 12 model genes along pseudotime. ****Means *P* < 0.0001; ***Means *P* < 0.001; **Means *P* < 0.01; *Means *P* < 0.05; ns Means not significant.

To explore the expression and distribution of PTM-related genes at single-cell resolution, we analyzed scRNA-seq data obtained from the GEO database. After standard quality control and data integration, a total of 22, 428 genes and 31, 916 cells were retained for downstream analysis (**Supplementary Figures 6a–c**). Subsequently, GO enrichment analysis based on the top 30 highly variable genes indicated that the development of PTC is associated with PTM, DNA repair, and immune-related pathways (**Supplementary Figure 7**). We defined six major cell types by combining automatic annotation using the ‘SingleR’ package with manual curation based on known marker genes (46)(**Figure 7a**), including normal thyroid follicular cells (TFCs), PTC cells, fibroblasts, endothelial cells, CD8+ T cells, and myeloid cells. To distinguish TFCs from PTC cells, we examined the expression of thyroid differentiation markers. As expected, TFCs exhibited a higher degree of differentiation (**Supplementary Figure 6d**). Marker genes for each cell type were presented in **Supplementary Figures 8**-**10**; **Figures 7b, c**. Moreover, analysis of cell type proportions and intercellular communication networks revealed that immune cells and fibroblasts are closely linked to PTC development and progression (**Figures 7d, e**; **Supplementary Figure 11**, **12**). Significant differences in PTMI were observed across cell types (**Figure 7f**), with PTC cells showing markedly higher PTMI levels (*P* < 2.2e-16). To further investigate cellular evolution and the dynamic regulation of model genes, we performed pseudotime trajectory analysis using the ‘monocle3’ package (**Figures 7g, h**; **Supplementary Figures 6e**, **13a–f**). The distribution and expression of the twelve model genes across different cell types were displayed in **Supplementary Figure 6f**. In summary, our single-cell analysis provided compelling evidence that PTMI is closely associated with PTC progression and demonstrates distinct cellular-level specificity. Meanwhile, owing to the limited number of single-cell samples from a single institution, these analyses are exploratory and require confirmation in larger and multicenter cohorts.

### PTMI correlates with the immune features of PTC patients

**Figure 8:**
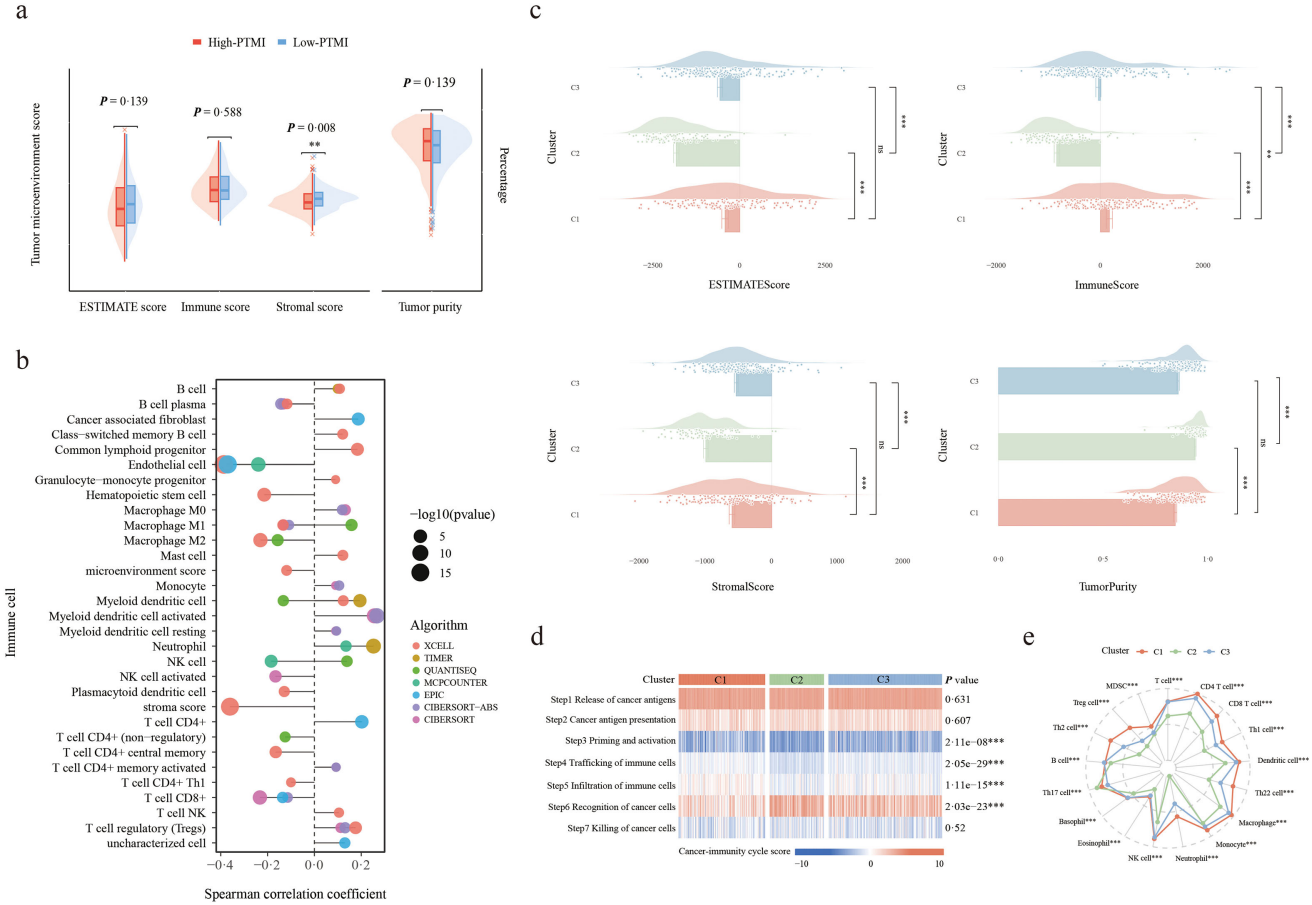
The immune landscape associated with PTMI in PTC. **(a)** Comparison of ESTIMATE scores between the PTMI subgroups. **(b)** The lollipop plot shows the Spearman correlation between immune cell infiltration and the PTMI. **(c)** Barplots show the ESTIMATE scores of PTMI clusters. **(d)** Heatmap demonstrating the difference in the seven-step anticancer immunity cycle activity among the three PTMI clusters. **(e)** The radar plot shows the difference in the ability of PTMI clusters to recruit immune cells. ***Means *P* < 0.001; **Means *P* < 0.01; *Means *P* < 0.05; ns Means not significant.

To explore the association between PTMI and TIME in PTC, we conducted comprehensive immune profiling analyses. Between different PTMI subgroups, we assessed the enrichment scores of immune-related components using the ESTIMATE algorithm (**Figure 8a**). The stromal score was significantly elevated in the high-PTMI group (*P* = 0.008). In terms of immune cell abundance, PTMI showed positive correlations with Cancer-Associated Fibroblasts (CAFs), myeloid dendritic cells, M0 macrophages, and neutrophils etc. In contrast, PTMI were negatively associated with anti-tumor immune cell types, including NK cells, CD4+ T cells, and CD8+ T cells (**Figure 8b**). Among the distinct clusters, we observed significant differences in the ESTIMATE score, immune score, stromal score, and tumor purity, with C1 displaying the most immunosuppressive profile (**Figure 8c**). To further dissect the functional impact of immune cells, we evaluated the activities of the seven steps in the anti-cancer immunity cycle. C1 and C3 exhibited increased activity in step 3 (priming and activation), step 4 (trafficking of immune cells), and step 5 (infiltration of immune cells), but reduced activity in step 6 (recognition of cancer cells) (**Figure 8d**). Additionally, we investigated Step 4 in greater detail by analyzing the recruitment patterns of various immune cell types (**Figure 8e**). BRAF-like tumors (C1) demonstrated the strongest recruitment capacity across most immune cell types. Notably, the recruitment of Treg cells and neutrophils increased with PTMI levels. These findings provide novel insights into the intricate interplay between PTMI and TIME reprogramming in PTC and the potential application of immunotherapy.

### Efficacy of the PTMI signature in predicting therapeutic response in PTC

**Figure 9:**
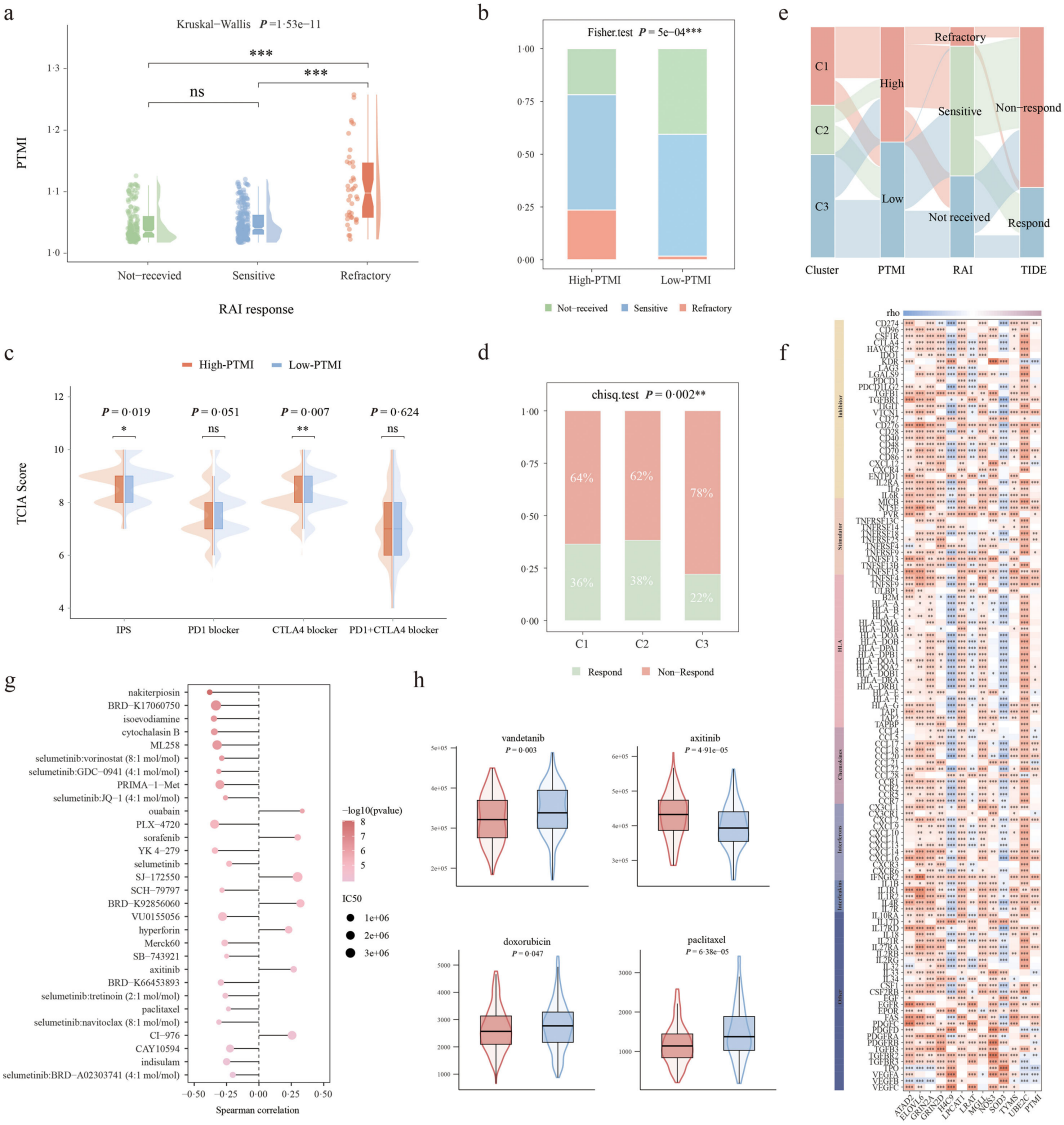
Prediction of multiple treatment responses based on PTMI features. **(a)** Boxplot depicting the differences of PTMI among the RAI treatment condition groups. **(b)** Distribution differences in objective RAI treatment responses between PTMI subgroups. **(c)** The TCIA algorithm predicted the probability of the response to PD1/PDL1 and CTLA4 blockers in the PTMI subgroups. **(d)** Distribution differences in putative ICI treatment responses derived from TIDE between PTMI clusters. **(e)** Alluvial diagram showing the interrelationship between the molecular clusters, PTMI subgroups, RAI, and ICI treatment responses in PTC patients. **(f)** Spearman correlation between immunomodulators (co-inhibitors, co-stimulators, and HLA molecules) and PTMI signatures. **(g)** Top 30 compounds (from the CTRP v2 database) with the most significant differences in predicted IC50 between PTMI subgroups. **(h)**The IC50 of MTT and CTx agents between PTMI subgroups. ***Means *P* < 0.001; **Means *P* < 0.01; *Means *P* < 0.05; ns Means not significant.

Given the pivotal value of PTMI in the diagnosis and prognosis of PTC, we further evaluated its potential utility in guiding treatment strategies. For RAI therapy, we compared the PTMI under different RAI conditions. PTMI levels were significantly different among the non-received, RS, and RR groups (***P* **= 1.53e-11, **Figure 9a**) and the AUC for PTMI in predicting RAI response was 0.789 (**Supplementary Figure 15E**). Notably, RR was defined by TCFi events after RAI, and TCFi is also what the PTMI predicts, so part of this association comes from the definition itself. We therefore treat **Figures 9a, b** as descriptive and do not interpret it as evidence that the PTMI predicts refractoriness. The high-PTMI group contained a greater proportion of PTC-RAI patients (70% vs. 59%) and a higher RR rate (15% vs. 2%) compared to the low-PTMI group (*P* = 5e-04; **Figure 9b**). For immunotherapy, we predicted the response to immune checkpoint inhibitors (ICIs) using the TCIA and TIDE algorithms (**Figures 9c, d**). Distinct PTMI subgroups and molecular clusters exhibited significant differences in immunophenoscore (IPS) and ICIs response. The alluvial diagram further illustrated that C1 corresponded to higher PTMI levels, a greater proportion of ICI responders, but fewer RAI responders (**Figure 9e**). Additionally, strong correlations were observed between PTMI signatures and multiple immunoregulatory molecules (**Figure 9f**). For drug sensitivity, we estimated the half maximal inhibitory concentration (IC50) values for a range of agents in PTC patients. A comprehensive landscape of the correlation between drug sensitivity and PTMI was shown in **Figures 9g, h**; **Supplementary Figure 16**. The high-PTMI group showed increased sensitivity to vandetanib, doxorubicin, and paclitaxel, among others, whereas the low-PTMI group demonstrated greater sensitivity to axitinib and lapatinib, etc. Notably, nakiterpiosin emerged as a promising candidate for the treatment of PTC at the advanced stage. These findings suggest that PTC patients with high PTMI were more likely to benefit from standard chemotherapeutic regimens. In brief, the PTMI signature serves as an ideal predictor of the response across multiple therapeutic modalities, offering new opportunities for personalized treatment strategy in PTC.

### Internal and external validation of PTMI signatures in PTC

**Figure 10:**
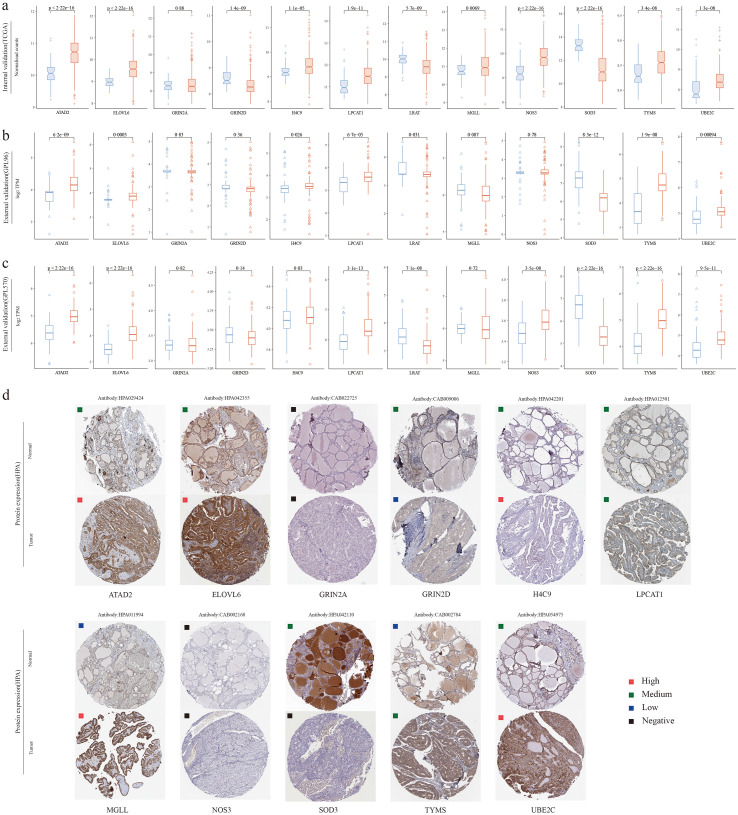
Internal and external validation of model genes in PTC. **(a)** Internal validation of the mRNA expression of 12 model genes between 477 PTC and 59 NT from the entire TCGA cohort. **(b)** External validation of the mRNA expression of 12 model genes between 113 PTCs and 53 NTs from the GEO database (GPL96 sets). **(c)** External validation of the mRNA expression of 12 model genes between 96 PTCs and 95 NTs from the GEO database (GPL570 sets). **(d)** Representative IHC staining images presenting the protein expression levels of model genes in NT and PTC tissues from the HPA database.

To further assess the clinical applicability of the PTMI signature, we evaluated the expression profiles of the 12 component genes from multiple dimensions. Internal validation was conducted using the entire TCGA cohort (**Figure 10a**), while the differential expression of PTMI signatures was replicated in two independent GEO datasets, GPL96 and GPL570 (**Figures 10b, c**). Most genes exhibited significant differential expression between PTC and NT tissues, and the observed trends were consistent with previous findings. Protein-level validation was further conducted via immunohistochemistry (IHC) (**Figure 10d**). Consistent with the mRNA expression level, ATAD2, ELOVL6, H4C9, MGLL, TYMS, and UBE2C showed pronounced upregulation in PTC tissues, whereas GRIN2D, NOS3, and SOD3 were markedly downregulated. In addition, expression of these genes was also profiled across multiple THCA cell lines, revealing heterogeneous expression patterns (**Supplementary Figure 17**).

### TYMS is a key functional PTMI signature associated with the malignant phenotypes of PTC

**Figure 11:**
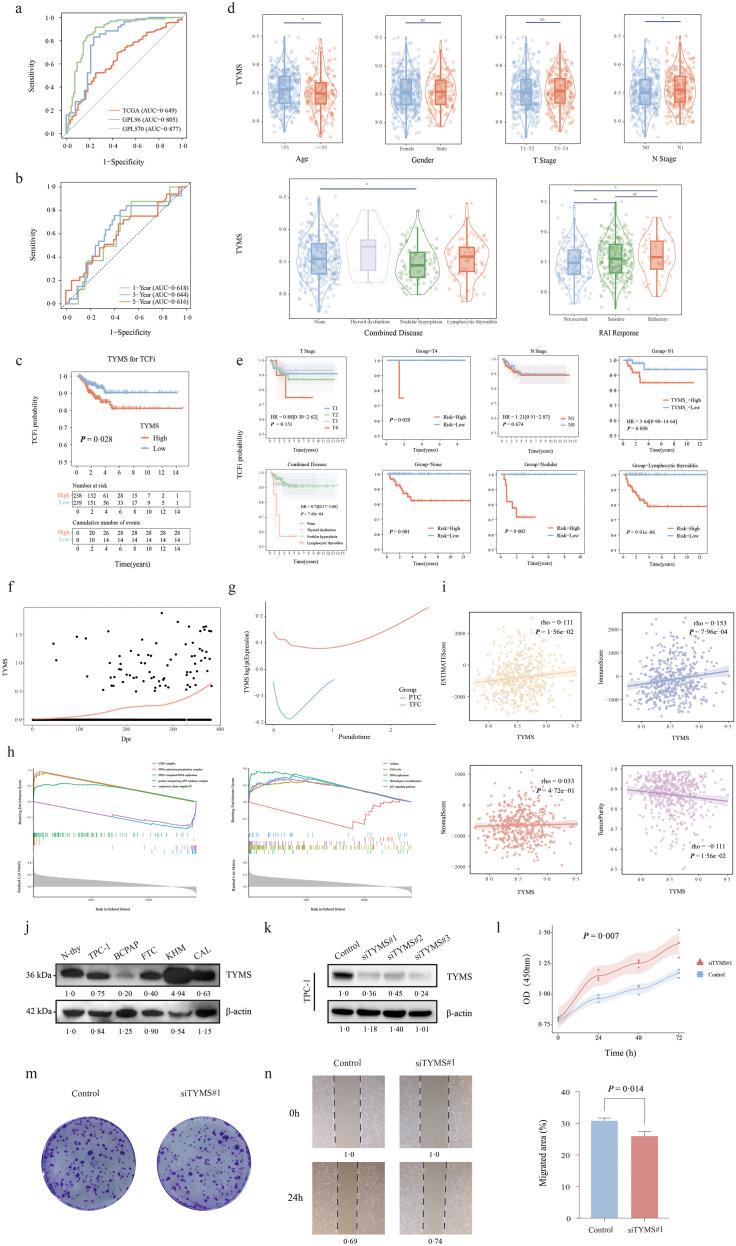
TYMS expression in PTC: implications for diagnosis, prognosis, and immune profiling. **(a, b)** ROC curves to evaluate the predictive ability of TYMS on diagnosis and prognosis in PTC patients. **(c)** TCFi in the low- and high-TYMS group patients. **(d)** Violin plots of the relationship between TYMS expression and Age, Gender, T stage, N stage, combined disease, and RAI response in PTC patients. **(e)** Kaplan-Meier analysis of the prognosis of PTC patients belonging to groups based on PTMI.**(f)** Line plot of TYMS expression along pseudotime in PTC cells. **(g)** The differential expression profiles of TYMS in PTC and TFC cells. **(h)** GSEA analysis (GO and KEGG) based on TYMS expression in PTC cells. **(i)** Association between the ESTIMATE scores and the TYMS expression. **(j)** The expression level of TYMS in normal thyroid cell lines (N-thy) and different PTC cell lines (TPC-1, BCPAP, FTC, KHM, and CAL). **(k)** Silencing effect of siRNA-TYMS. **(l)** The effect of siTYMS on the proliferation of TPC-1 cells measured by CCK-8. **(m, n)** The effect of siTYMS on the proliferation ability and migration ability of TPC-1 cells was measured by colony formation assay and wound-healing assay. Below the picture are the relative quantification values, the ratios normalized to the control group. All *in vitro* experiments were performed in three independent biological replicates. The CCK-8 data were analyzed by the two-tailed Student’s t-test and the wound-healing data by one-way ANOVA. Results are stated as mean ± SD. **Means *P* < 0.01; *Means *P* < 0.05; ns Means not significant.

To identify the core gene driving PTC progression among the PTMI signatures, we conducted a series of integrated analyses. We first assessed their diagnostic and prognostic value. ROC analysis (**Figures 11a, b**; **Supplementary Figure 18**) and K-M curves (**Figure 11c**; **Supplementary Figure 19**) demonstrated that TYMS had a superior performance in both diagnostic classification and prognostic prediction for PTC. Further analysis showed that TYMS effectively distinguished multiple clinical subgroups, particularly concerning age, N stage, combined disease, and RAI response (**Figure 11d**). Moreover, TYMS retained strong predictive performance across these clinical categories (**Figure 11e**). To explore its dynamic expression profile, we employed single-cell RNA sequencing analysis. TYMS expression was significantly higher in PTC cells compared to TFCs and increased progressively along the pseudotime trajectory (**Figures 11f, g**; **Supplementary Figure 14**). Besides, GSEA analysis revealed that TYMS may contribute to PTC progression by regulating pathways involved in DNA replication, the p53 signaling pathway, and the cell cycle (**Figure 11h**). Additionally, TYMS expression was positively correlated with the ESTIMATE score (rho=0.111, *P* = 1.56e-02) and immune score (rho=0.153, *P* = 7.96e-04), and negatively correlated with tumor purity (rho=-0.111, *P* = 1.56e-02) (**Figure 11i**). Collectively, these findings highlight TYMS as a key functional component of the PTMI signature, playing an essential role in regulating the malignant phenotypes of PTC.

Furthermore, as the key factor in regulating the malignant phenotypes of PTC, we validated the role of TYMS *in vitro* experiments. We assessed the expression of TYMS in multiple human PTC cell lines and normal thyroid cell lines (**Figure 11j**). TYMS expression was heterogeneous across the thyroid cancer cell lines, with markedly higher expression in KHM, an anaplastic thyroid carcinoma (ATC) cell line that is biologically distinct from the PTC cell lines (TPC-1, BCPAP). This pattern is consistent with the high proliferative activity of ATC and indicates that TYMS expression varies across thyroid cancer subtypes. We therefore do not claim uniform TYMS overexpression, and the subtype-specific expression of KHM warrants further investigation. In view of its high expression, we constructed TYMS-knockdown PTC cell models using siRNA to investigate its regulatory effects (**Figure 11k**). Experimental results indicated that silencing TYMS markedly impeded the proliferation (**Figures 11l, m**) and migration ability of PTC cells (**Figure 11n**). These findings demonstrated that TYMS may act a crucial role in modulating the malignant progression of PTC.

## Discussion

Our study systematically investigated the role of PTMs in the development and progression of PTC. We first characterized 17 distinct PTMs and subsequently established a consensus prognostic PTMI using multiple machine learning algorithms. Incorporating clinicopathological variables, we further evaluated SHAP and nomogram values to assess the clinical utility of PTMI, which confirmed its robust and superior performance. Notably, PTMI showed a significant association with TIME remodeling and provided novel insights into the prediction of therapeutic responses in PTC. In addition, we also identified the core gene driving PTC progression among the PTMI signatures. Together, these findings emphasize the potential clinical applications of the PTMI in guiding personalized treatment decisions.

Increasing evidence indicates that PTMs play a fundamental role in biological and pathological processes, which have been verified to be related to the development and metastasis of malignant tumors for decades (47–49). Herein, we constructed a signature comprising 12 PTM-DEGs (ATAD2, ELOVL6, GRIN2A, GRIN2D, H4C9, LPCAT1, LRAT, MGLL, NOS3, SOD3, TYMS, and UBE2C). Considering the indolent characteristics of PTC, we proposed to select TCFi as the prognostic indicator for higher disease specificity and predictive value. PTMI exhibits good predictive performance for TCFi in PTC. Then we conducted a comprehensive bioinformatic analysis to investigate the genetic landscape and clinical relevance of these model genes in PTC.

Among the 12 model genes, six oncogenes (ATAD2, H4C9, LPCAT1, MGLL, TYMS, and UBE2C) were upregulated in PTC and associated with poor prognosis. Notably, TYMS emerged as the core gene driving tumor progression. TYMS was prioritized not for its fold change but for convergent evidence, including diagnostic and prognostic ROC, survival stratification, progressive up-regulation along the single-cell pseudotime trajectory, enrichment in DNA replication, p53 and cell-cycle pathways, and the *in vitro* finding that its knockdown reduced PTC cell proliferation and migration. TYMS catalyzes the conversion of dUMP to dTMP and is the classical target of antifolate agents such as 5-fluorouracil. TYMS also supports the nucleotide supply required for telomere maintenance (50). In thyroid cancer, TYMS has recurred in prognostic gene signatures across papillary, differentiated, and anaplastic disease (51–53), yet it has rarely been studied directly. To our knowledge, its function has not been examined experimentally in PTC. This gap was a further reason for prioritizing TYMS, and our knockdown experiments provide initial functional evidence for its role. In contrast, SOD3 was downregulated in PTC and associated with favorable prognosis, suggesting its tumor-suppressive role. Interestingly, ELOVL6 and NOS3 were highly expressed in tumor tissues but linked to better clinical outcomes, indicating a paradoxical role in promoting tumorigenesis while restraining progression. Conversely, GRIN2D and LRAT were expressed at lower levels in tumor tissues but unexpectedly linked to poor clinical outcomes. Their adverse prognostic values appeared to be mediated through E2F and JAK2/STAT5 signaling pathways (54, 55). Additionally, GRIN2A seemed to participate in tumor initiation by modulating calcium channel signaling acting as a protective factor (56). As a multivariable risk score, the genes carry variable-importance values of both positive and negative signs. Because the PTMI is the risk predicted by the model rather than a linear sum, the contribution of any single gene is not directly recoverable from the score.

To assess the clinical applicability of PTMI in PTC, we developed SHAP and nomogram models by integrating PTMI with clinicopathological variables. In the SHAP model for TCFi prediction, PTMI exhibited the highest feature importance. Furthermore, the nomogram demonstrated superior discrimination ability and net benefit than the ATA, TNM, MACIS, and EORTC staging systems. These findings underscore the potential of PTMI as a clinical tool for personalized prognostic assessment.

Single-cell analysis and functional analysis revealed the underlying mechanisms in PTC progression, including PTMs, DNA repair processes, and immune-related pathways. To further elucidate the relationship between PTMI and TIME, we systematically compared TIME characteristics across PTMI subgroups and molecular clusters. The high-PTMI group displayed markedly reduced infiltration of anti-tumor immune cells, including NK cells, CD4+ T cells, and CD8+ T cells. In contrast, immunosuppressive cell populations such as CAFs, myeloid dendritic cells, M0 macrophages, and neutrophils were significantly enriched in the high-PTMI group. Additionally, we also uncovered substantial activation of checkpoint, HLA, and co-inhibition pathways in the high-PTMI group. These findings suggest that the high-PTMI group possessed a more immunosuppressive phenotype, characterized by pronounced immune evasion (57, 58).

A broader understanding of the interplay between PTC progression and the TIME may pave the way for novel therapeutic approaches targeting aggressive PTC. Surgical resection remains the primary and dominant treatment for PTC, with most patients achieving no evidence of structural disease following initial therapy (59). However, for those presenting with advanced-stage disease and aggressive histological subtypes, individualized treatment strategies beyond surgery are warranted to improve clinical outcomes. PTMI demonstrated strong predictive potential for treatment response across multiple therapeutic modalities, including RAI therapy, immunotherapy, and pharmacotherapy. In terms of RAI response, the high-PTMI group exhibited a higher proportion of the RR group. Previous evidence supports the role of the transamidase complex in post-translational regulation during tumorigenesis and its critical involvement in iodine uptake and retention in THCA (60). However, radioactive iodine therapy was administered at the clinician’s discretion rather than by randomization. As a result, the patients who received it had more advanced disease at baseline than those who did not, with higher T, N, and M categories, more aggressive histology, higher ATA risk, and higher PTMI (**Supplementary Table 2**). This baseline imbalance may confound analyses that relate RAI treatment or RAI response to outcome, and such results should be interpreted with caution. For immunotherapy, predictions generated by the TIDE and TCIA algorithms further confirm that the high-PTMI group may derive greater benefit from ICIs, particularly the CTLA-4 blocker. For drug sensitivity, several kinase inhibitors approved by the FDA have emerged as highly promising treatments for advanced DTC, including sorafenib, axitinib, and vandetanib (36, 61, 62). In our analysis, vandetanib demonstrated higher efficacy in the high-PTMI group, while axitinib showed greater effectiveness in the low-PTMI group. Moreover, the high-PTMI group exhibited increased sensitivity to conventional chemotherapeutics such as doxorubicin and paclitaxel. We also identified nakiterpiosin as a potential therapeutic agent with higher predicted efficacy in the high-PTMI group. Notably, nakiterpiosin may be an alternative chemotherapeutic agent against tumors resistant to antitubulin agents and reliant on Hedgehog signaling. Collectively, these findings highlight PTMI as a robust biomarker for guiding personalized treatment decisions and optimizing therapeutic strategies in PTC management (63).

Although the PTMI demonstrated robust performance in both the training and testing sets, it is important to acknowledge several limitations. Initially, to assess optimism arising from screening outside the cross-validation folds, we additionally performed a nested ten-fold cross-validation within the training set, which yielded a C-index of 0.604. Subsequently, the analyses were predominantly based on retrospective datasets, underscoring the need for prospective studies to validate the clinical utility and generalizability of PTMI. As prognostic information for PTC is available only in the TCGA-THCA dataset currently, external validation of the prognostic performance of the PTMI in an independent cohort with adequate follow-up remains an unmet need. And in the GEO cohorts, technical variance was large before correction (PC1 = 83.9% and 93.8% for GPL96 and GPL570, respectively). Although batch correction successfully intermixed the datasets, a correction of this magnitude may also have removed some biologically meaningful variation. The differential expression results from the GEO platforms should therefore be interpreted with caution, and replication in a platform-homogeneous cohort with lower inter-dataset technical variance would further strengthen these findings. Due to the limited number of single-cell samples from a single institution, these analyses are exploratory and require confirmation in larger and multicenter cohorts. And the RAI-refractory definition used here is partly circular, as TCGA lacks the imaging and biochemical data required for an independent ATA-based classification. The immunotherapy and drug sensitivity results were computationally predicted and not validated clinically. The results are presented as exploratory and potential findings that require experimental and clinical confirmation. We are assembling a prospectively followed cohort to address these in future work. Although internal validation was performed through random sample splitting, it is still necessary to verify the reliability of PTMI using multicenter randomized controlled trials that include more high-quality, large-scale samples with adequate follow-up periods. Meanwhile, independent STR authentication and mycoplasma testing of the cell lines were not performed, which we also acknowledge as a limitation of the study.

## Conclusion

In summary, our findings provide proof of concept that PTMs are closely associated with the development and progression of PTC. The PTMI proposed in this study demonstrates strong potential as a prognostic biomarker and therapeutic predictor, offering valuable insights for personalized management of PTC patients. Moreover, TYMS may be a key driver of PTC malignant progression.

## Data Availability

The datasets analyzed in this study are publicly available from The Cancer Genome Atlas (TCGA) and the Gene Expression Omnibus (GEO). The code for PTMI construction and all downstream analyses, together with the processed data supporting the findings, has been deposited in a public repository (GitHub: https://github.com/WKY0624/PTMI-PTC). The experimental data related to TYMS silencing are presented in the Results section.
