# Supplementary material for: Integrative profiling of diverse post-translational modifications for prognostic stratification and personalized therapy in papillary thyroid cancer
Source: Front Endocrinol (Lausanne). 2026 Jul 27;17:1875494. doi: 10.3389/fendo.2026.1875494 (PMC13454233; doi:10.3389/fendo.2026.1875494)
Supplement: Supplementary file 1 [file DataSheet1.pdf]

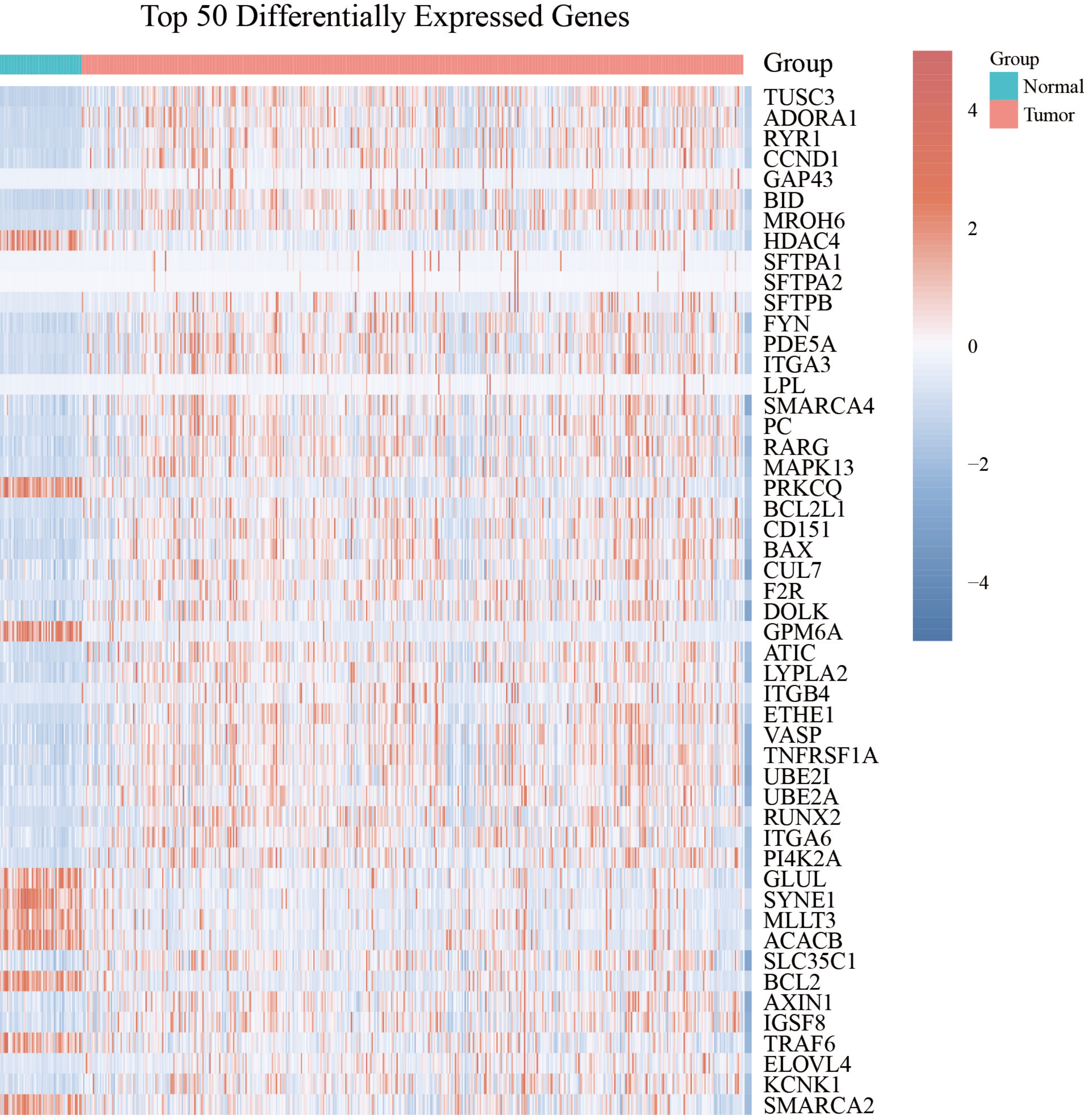
Top 50 Differentially Expressed Genes
Group
TUSC3
ADORA1
RYR1
CCND1
GAP43
BID
MROH6
HDAC4
SFTPA1
SFTPA2
SFTPB
FYN
PDE5A
ITGA3
LPL
SMARCA4
PC
RARG
MAPK13
PRKCQ
BCL2L1
CD151
BAX
CUL7
F2R
DOLK
GPM6A
ATIC
LYPLA2
ITGB4
ETHE1
VASP
TNFRSF1A
UBE2I
UBE2A
RUNX2
ITGA6
PI4K2A
GLUL
SYNE1
MLLT3
ACACB
SLC35C1
BCL2
AXIN1
IGSF8
TRAF6
ELOVL4
KCNK1
SMARCA2
4
2
0
−2
−4
Group
Normal
Tumor

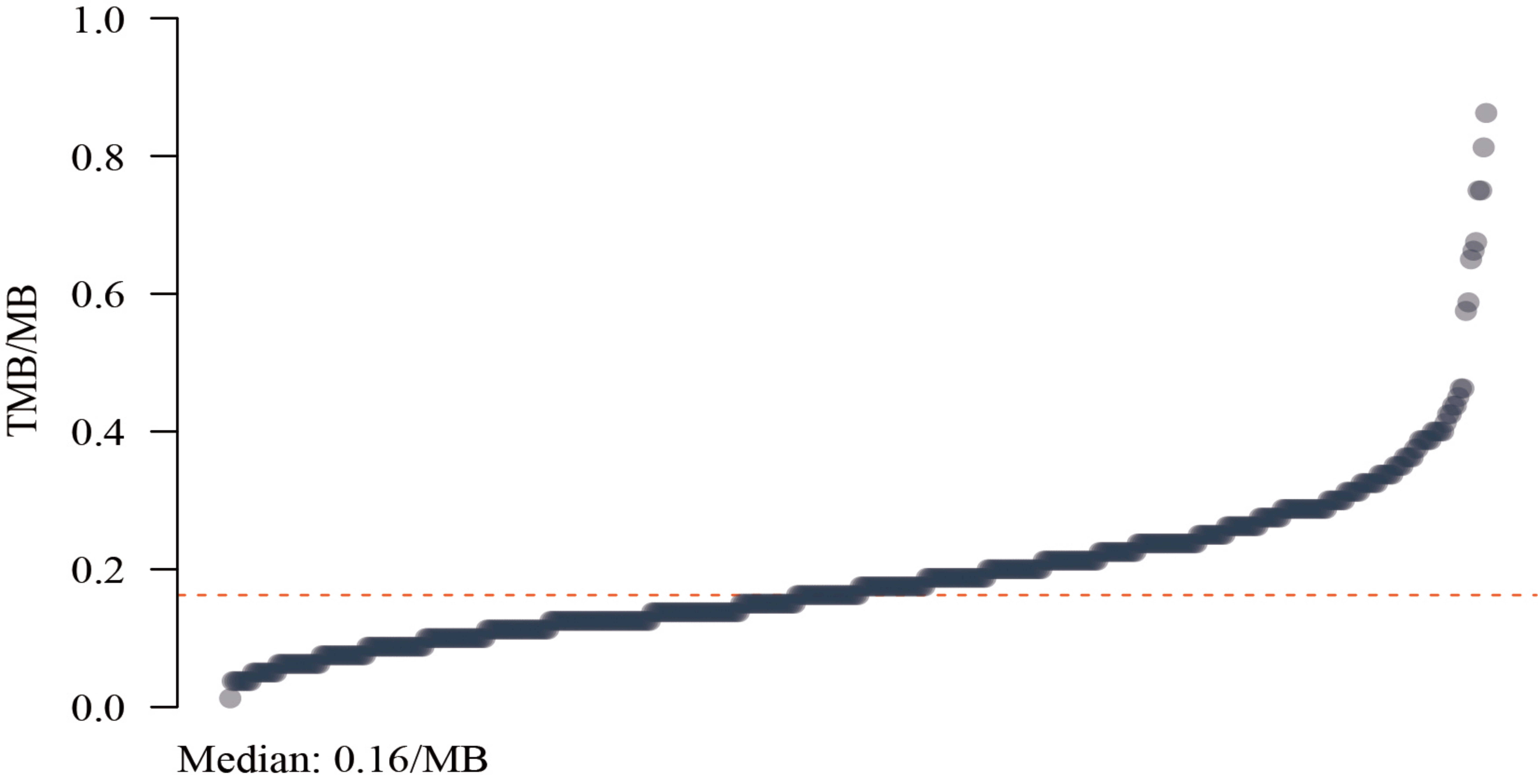

1.0
0.8
0.6
0.4
0.2
0.0
TMB/MB
Median: 0.16/MB

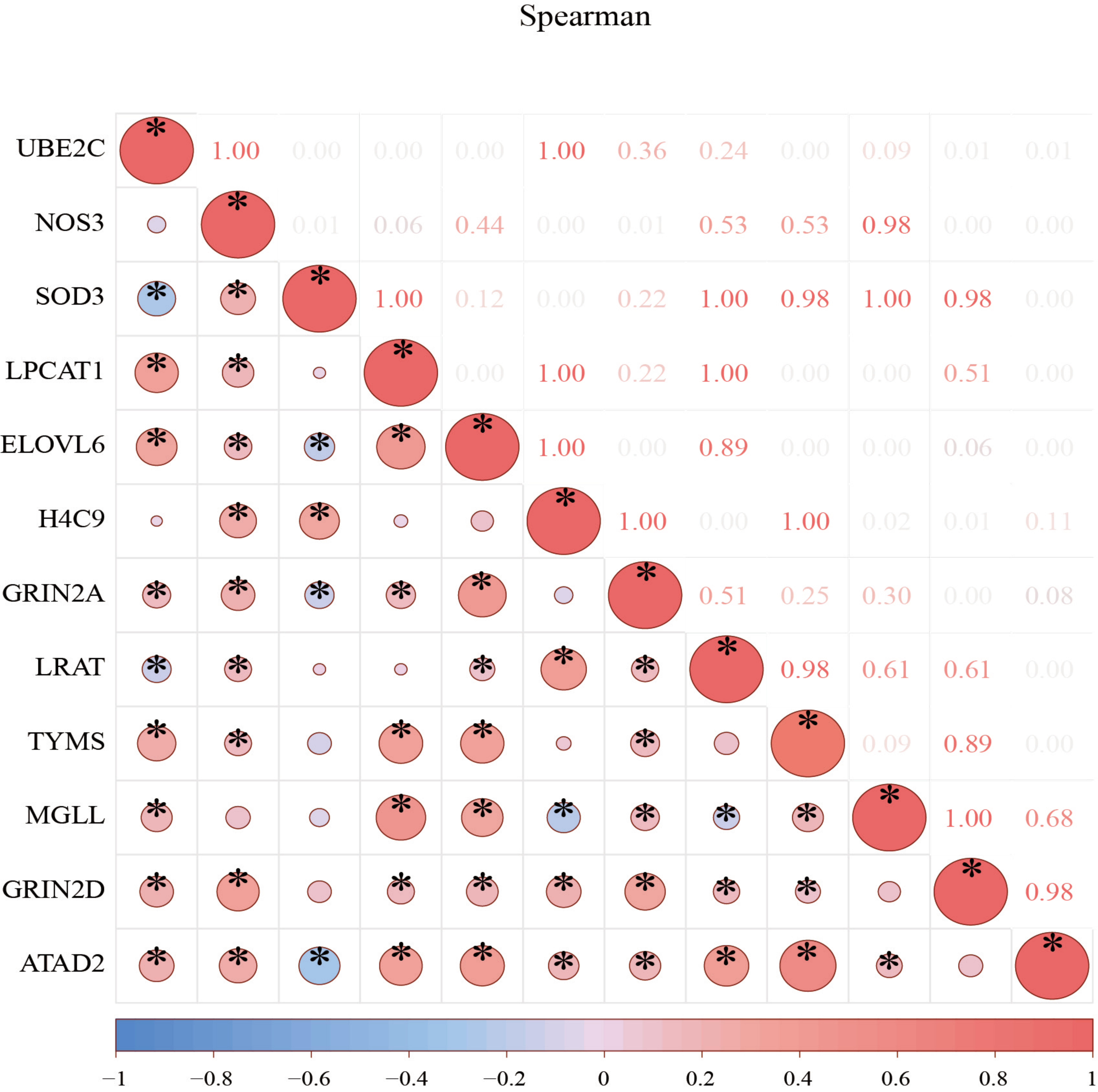
Spearman
UBE2C
NOS3
SOD3
LPCAT1
ELOVL6
H4C9
GRIN2A
LRAT
TYMS
MGLL
GRIN2D
ATAD2
1.00 0.00 0.00 0.00 1.00 0.36 0.24 0.00 0.09 0.01 0.01
0.01 0.06 0.44 0.00 0.01 0.53 0.53 0.98 0.00 0.00
1.00 0.12 0.00 0.22 1.00 0.98 1.00 0.98 0.00
0.00 1.00 0.22 1.00 0.00 0.00 0.51 0.00
1.00 0.00 0.89 0.00 0.00 0.06 0.00
1.00 0.00 1.00 0.02 0.01 0.11
0.51 0.25 0.30 0.00 0.08
0.98 0.61 0.61 0.00
0.09 0.89 0.00
1.00 0.68
0.98
−1
−0.8
−0.6
−0.4
−0.2
0
0.2
0.4
0.6
0.8
1

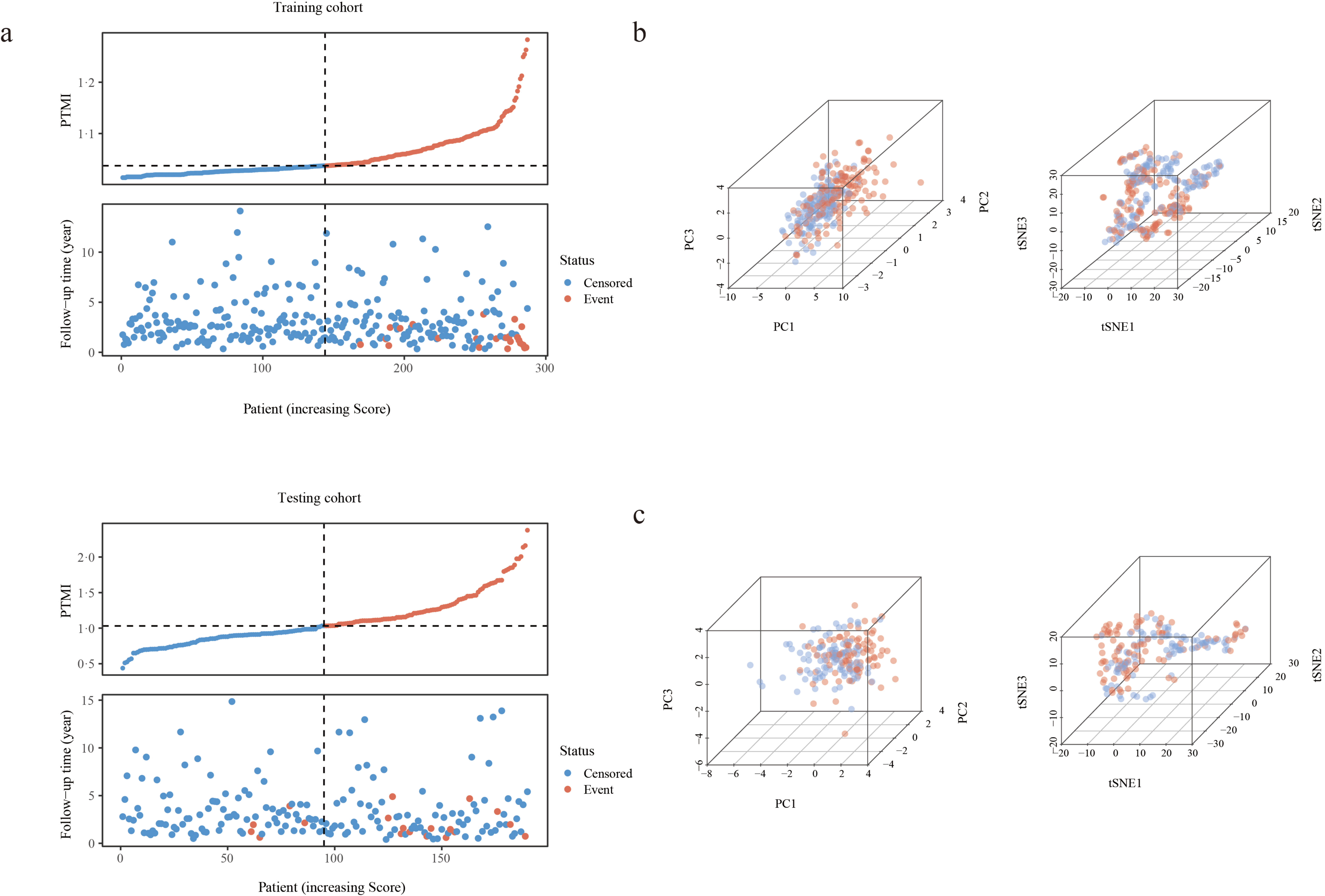

a
Training cohort
PTMI
Follow−up time (year)
Status
Censored
Event
Patient (increasing Score)
Testing cohort
PTMI
Follow−up time (year)
Status
Censored
Event
Patient (increasing Score)
b
PC1
PC2
PC3
tSNE1
tSNE2
tSNE3
c
PC1
PC2
PC3
tSNE1
tSNE2
tSNE3

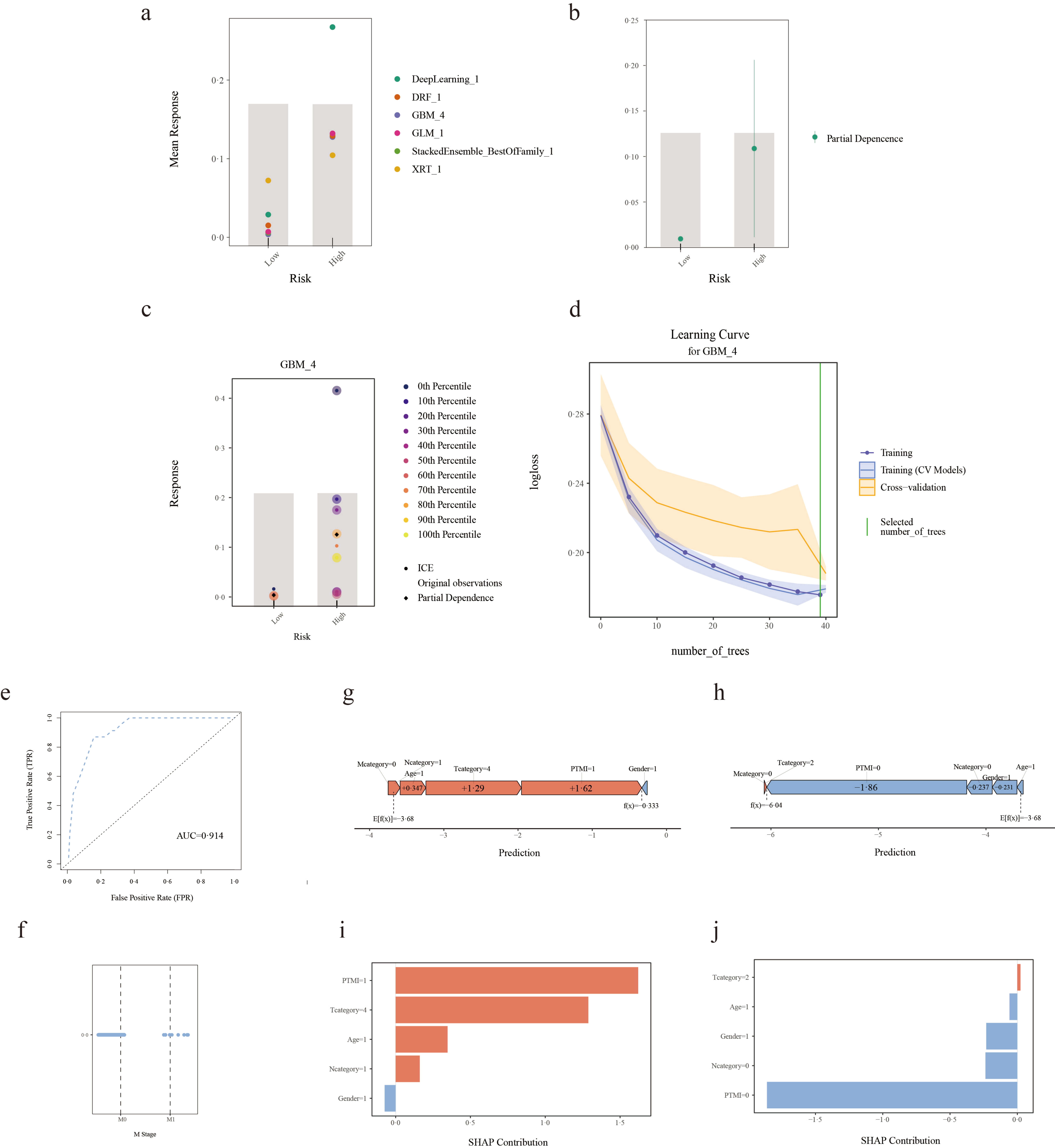

a
Mean Response
0·2
0·1
0·0
Low
High
Risk
DeepLearning_1
DRF_1
GBM_4
GLM_1
StackedEnsemble_BestOfFamily_1
XRT_1
b
0·25
0·20
0·15
0·10
0·05
0·00
Low
High
Risk
Partial Depencence
c
GBM_4
Response
0·4
0·3
0·2
0·1
0·0
Low
High
Risk
0th Percentile
10th Percentile
20th Percentile
30th Percentile
40th Percentile
50th Percentile
60th Percentile
70th Percentile
80th Percentile
90th Percentile
100th Percentile
ICE
Original observations
Partial Dependence
d
Learning Curve
for GBM_4
logloss
0·28
0·24
0·20
0
10
20
30
40
number_of_trees
Training
Training (CV Models)
Cross−validation
Selected
number_of_trees
e
True Positive Rate (TPR)
1·0
0·8
0·6
0·4
0·2
0·0
0·0
0·2
0·4
0·6
0·8
1·0
False Positive Rate (FPR)
AUC=0·914
g
Mcategory=0
Ncategory=1
Age=1
Tcategory=4
PTMI=1
Gender=1
+0·347
+1·29
+1·62
E[f(x)]=−3·68
f(x)=−0·333
−4
−3
−2
−1
0
Prediction
h
Mcategory=0
Tcategory=2
PTMI=0
Ncategory=0
Gender=1
Age=1
−1·86
−0·237
−0·231
f(x)=−6·04
E[f(x)]=−3·68
−6
−5
−4
Prediction
f
0·0
M0
M1
M Stage
i
PTMI=1
Tcategory=4
Age=1
Ncategory=1
Gender=1
0·0
0·5
1·0
1·5
SHAP Contribution
j
Tcategory=2
Age=1
Gender=1
Ncategory=0
PTMI=0
−1·5
−1·0
−0·5
0·0
SHAP Contribution

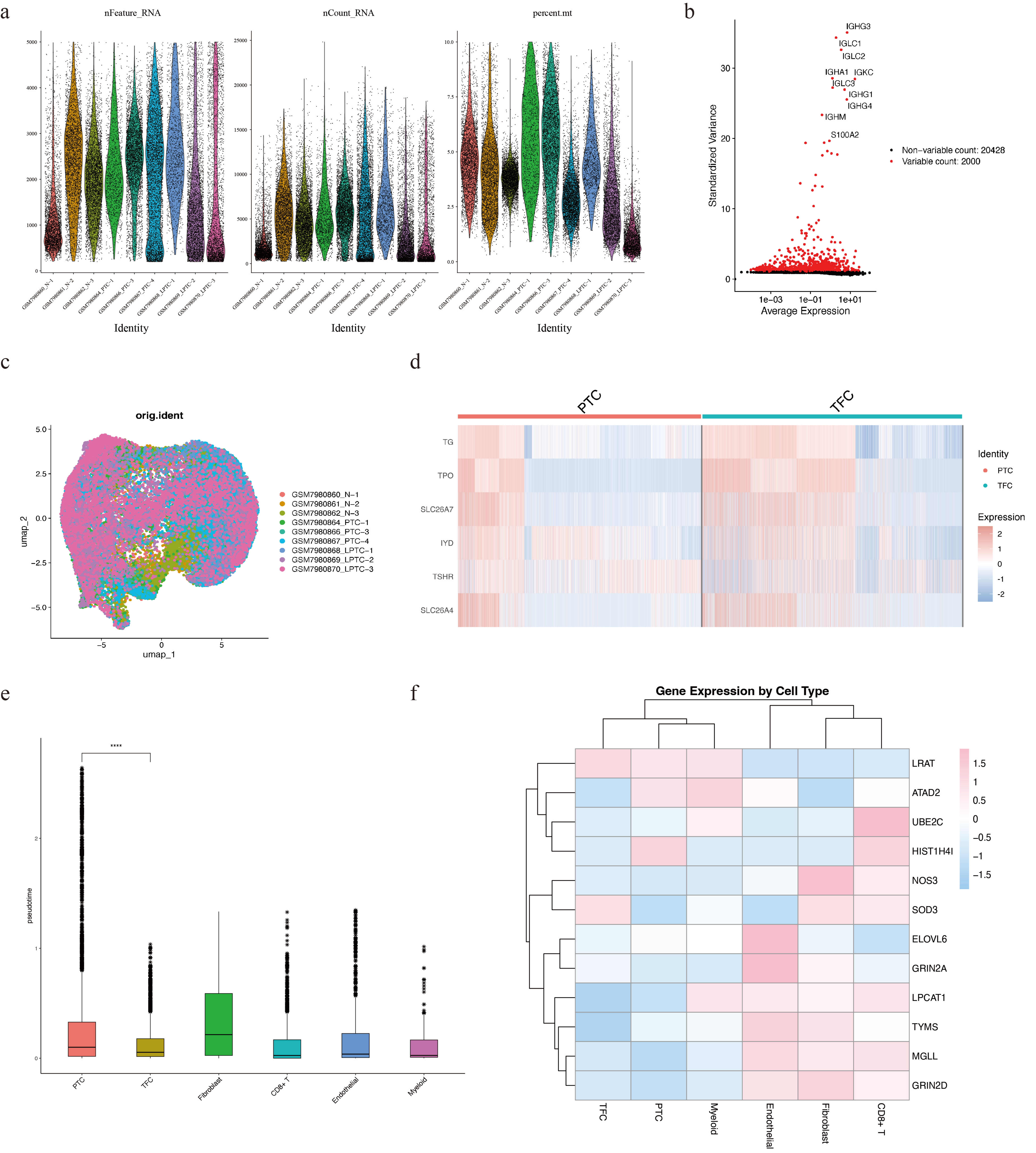
a
nFeature_RNA
nCount_RNA
percent.mt
Identity
GSM7980860_N-1
GSM7980861_N-2
GSM7980862_N-3
GSM7980864_PTC-1
GSM7980866_PTC-3
GSM7980867_PTC-4
GSM7980868_LPTC-1
GSM7980869_LPTC-2
GSM7980870_LPTC-3
b
IGHG3
IGLC1
IGLC2
IGHA1
IGKC
IGLC3
IGHG1
IGHG4
IGHM
S100A2
Standardized Variance
Average Expression
Non-variable count: 20428
Variable count: 2000
c
orig.ident
umap_1
umap_2
d
PTC
TFC
TG
TPO
SLC26A7
IYD
TSHR
SLC26A4
Identity
Expression
e
pseudotime
****
PTC
TFC
Fibroblast
CD8+ T
Endothelial
Myeloid
f
Gene Expression by Cell Type
LRAT
ATAD2
UBE2C
HIST1H4I
NOS3
SOD3
ELOVL6
GRIN2A
LPCAT1
TYMS
MGLL
GRIN2D

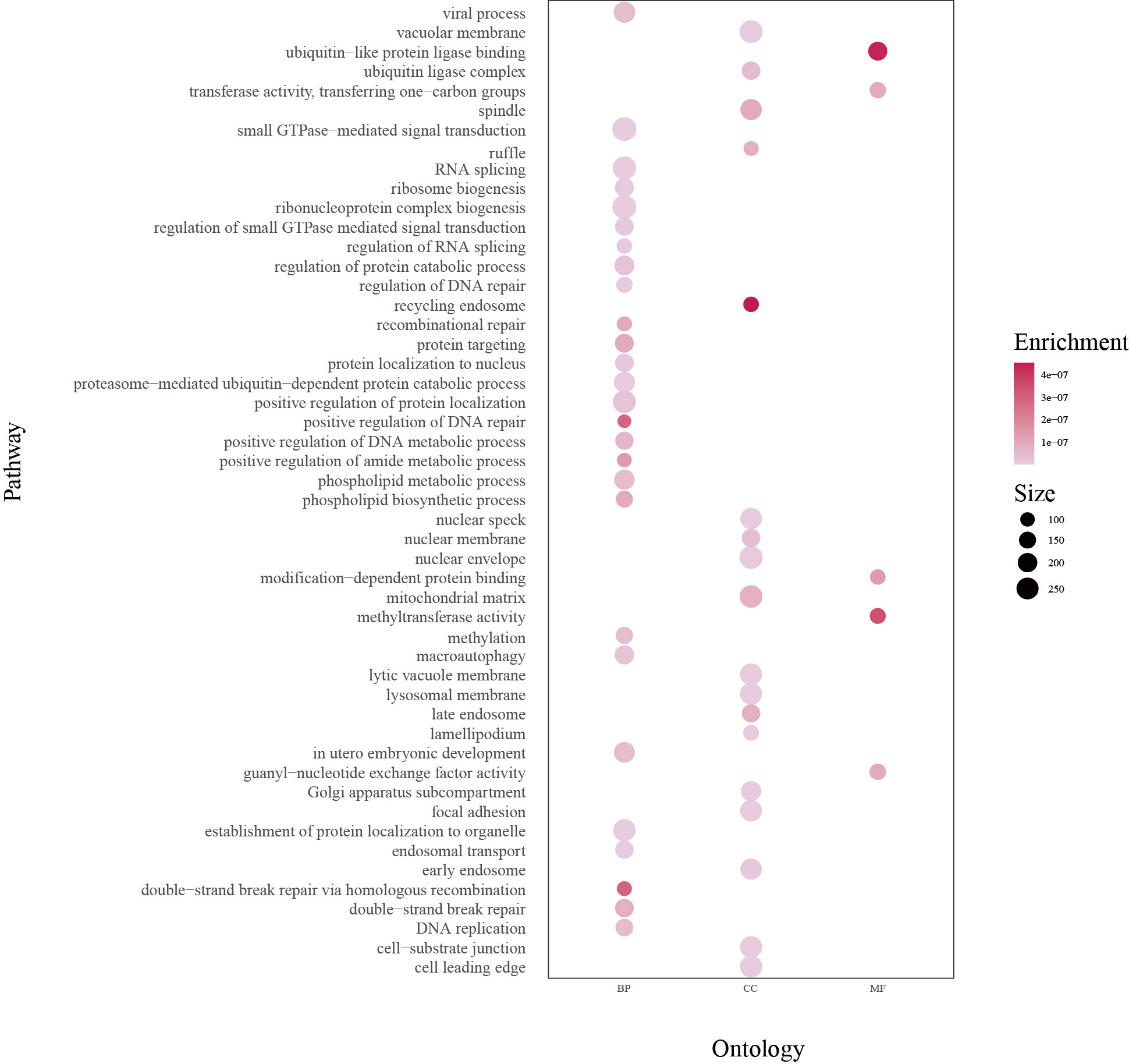

viral process
vacuolar membrane
ubiquitin−like protein ligase binding
ubiquitin ligase complex
transferase activity, transferring one−carbon groups
spindle
small GTPase−mediated signal transduction
ruffle
RNA splicing
ribosome biogenesis
ribonucleoprotein complex biogenesis
regulation of small GTPase mediated signal transduction
regulation of RNA splicing
regulation of protein catabolic process
regulation of DNA repair
recycling endosome
recombinational repair
protein targeting
protein localization to nucleus
proteasome−mediated ubiquitin−dependent protein catabolic process
positive regulation of protein localization
positive regulation of DNA repair
positive regulation of DNA metabolic process
positive regulation of amide metabolic process
phospholipid metabolic process
phospholipid biosynthetic process
nuclear speck
nuclear membrane
nuclear envelope
modification−dependent protein binding
mitochondrial matrix
methyltransferase activity
methylation
macroautophagy
lytic vacuole membrane
lysosomal membrane
late endosome
lamellipodium
in utero embryonic development
guanyl−nucleotide exchange factor activity
Golgi apparatus subcompartment
focal adhesion
establishment of protein localization to organelle
endosomal transport
early endosome
double−strand break repair via homologous recombination
double−strand break repair
DNA replication
cell−substrate junction
cell leading edge
Pathway
BP
CC
MF
Ontology
Enrichment
4e−07
3e−07
2e−07
1e−07
Size
100
150
200
250

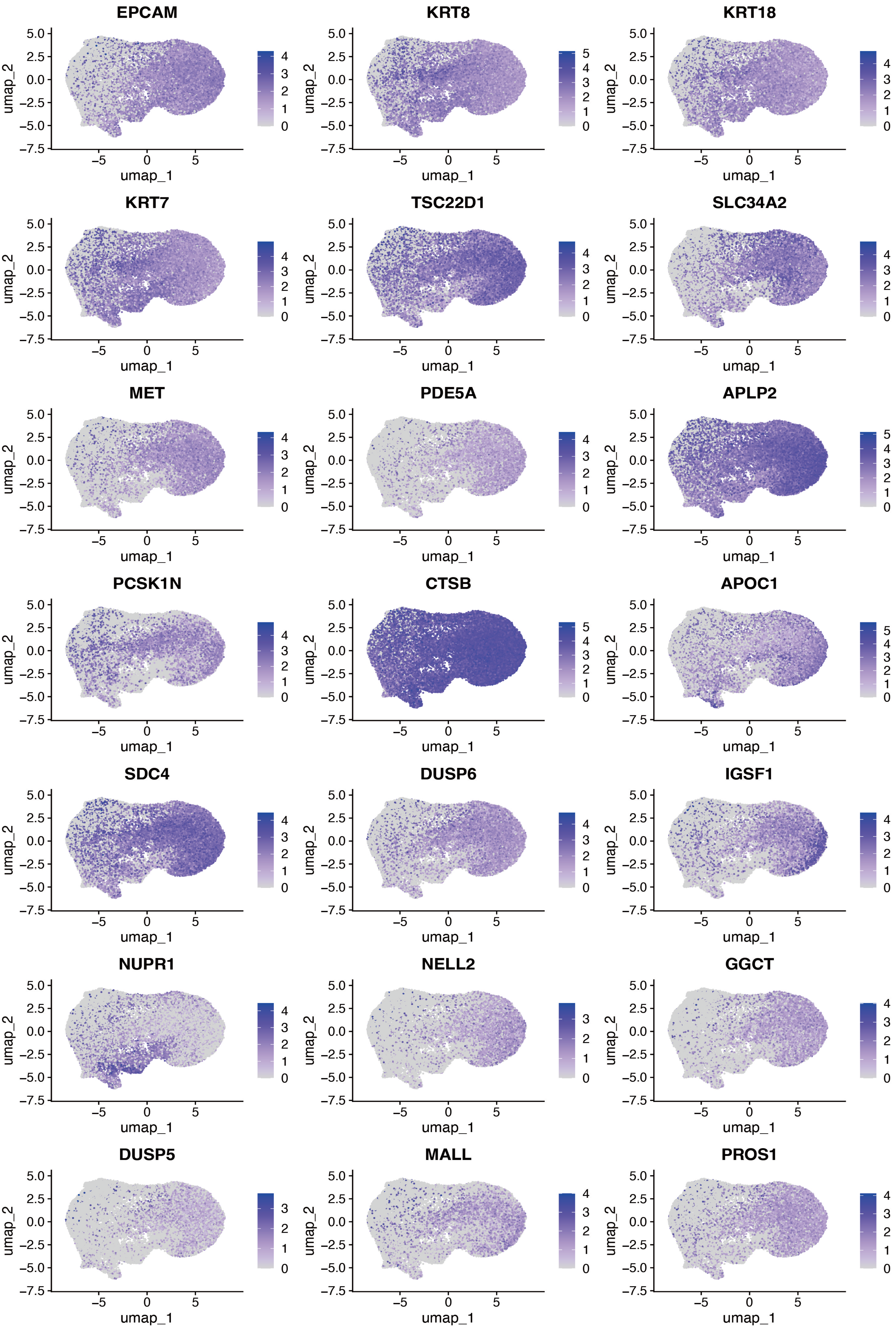
EPCAM
KRT8
KRT18
KRT7
TSC22D1
SLC34A2
MET
PDE5A
APLP2
PCSK1N
CTSB
APOC1
SDC4
DUSP6
IGSF1
NUPR1
NELL2
GGCT
DUSP5
MALL
PROS1
umap_1
umap_2
