# Supplementary material for: Integrative profiling of diverse post-translational modifications for prognostic stratification and personalized therapy in papillary thyroid cancer
Source: Front Endocrinol (Lausanne). 2026 Jul 27;17:1875494. doi: 10.3389/fendo.2026.1875494 (PMC13454233; doi:10.3389/fendo.2026.1875494)
Supplement: Supplementary file 2 [file DataSheet2.pdf]

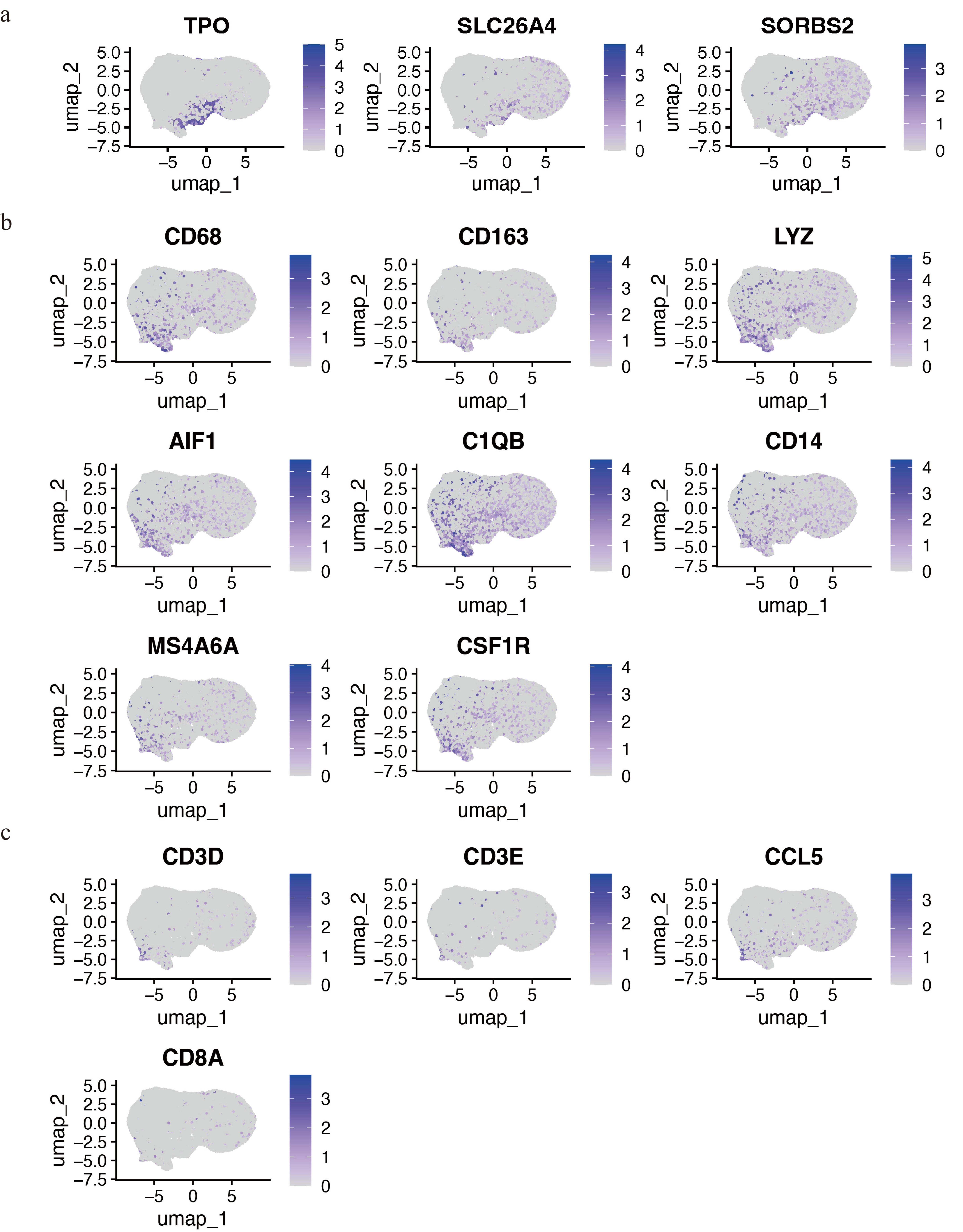
a
TPO
SLC26A4
SORBS2
b
CD68
CD163
LYZ
AIF1
C1QB
CD14
MS4A6A
CSF1R
c
CD3D
CD3E
CCL5
CD8A
umap_1
umap_2

a

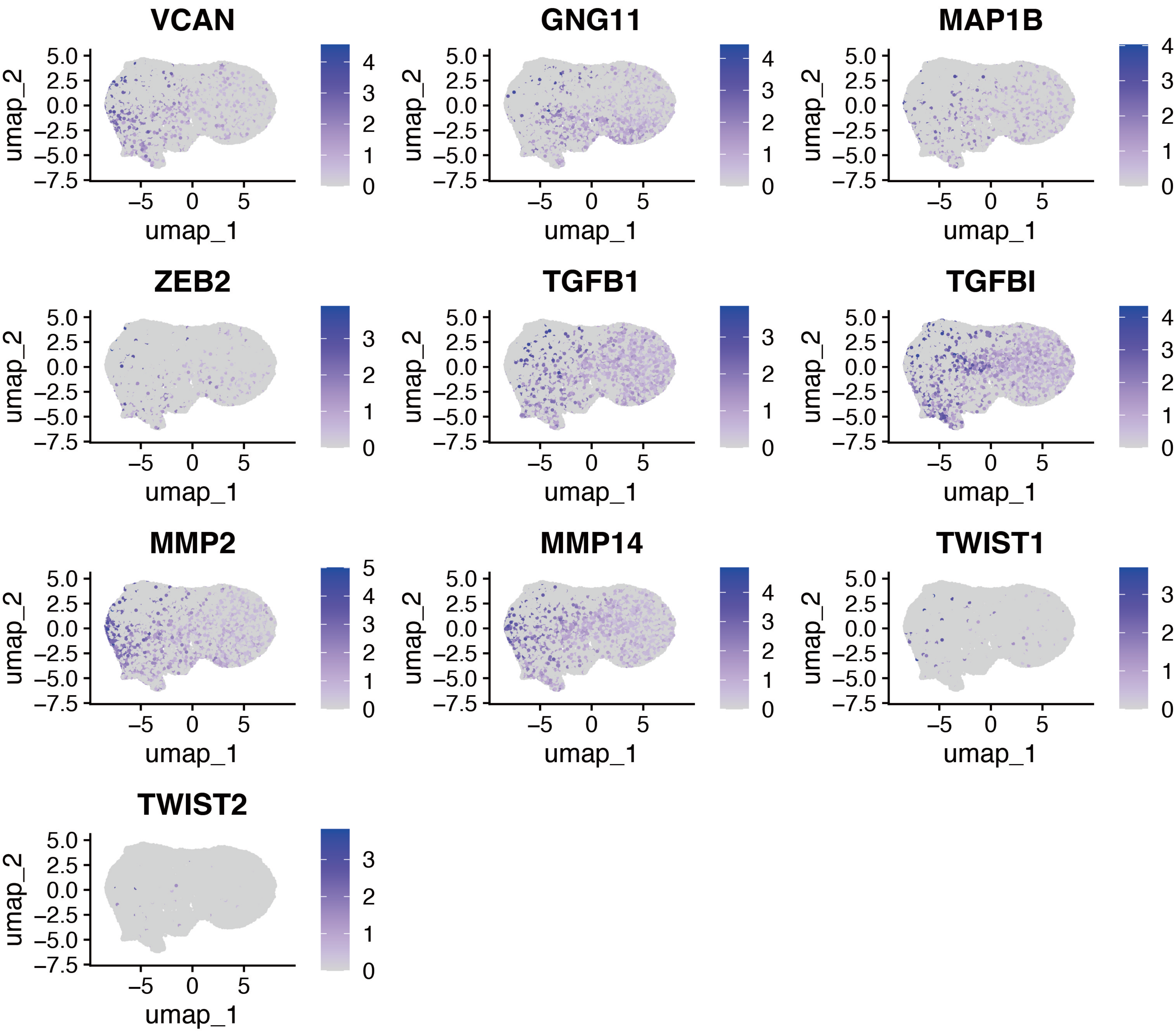
VCAN
GNG11
MAP1B
ZEB2
TGFB1
TGFBI
MMP2
MMP14
TWIST1
TWIST2
umap_1
umap_2

b

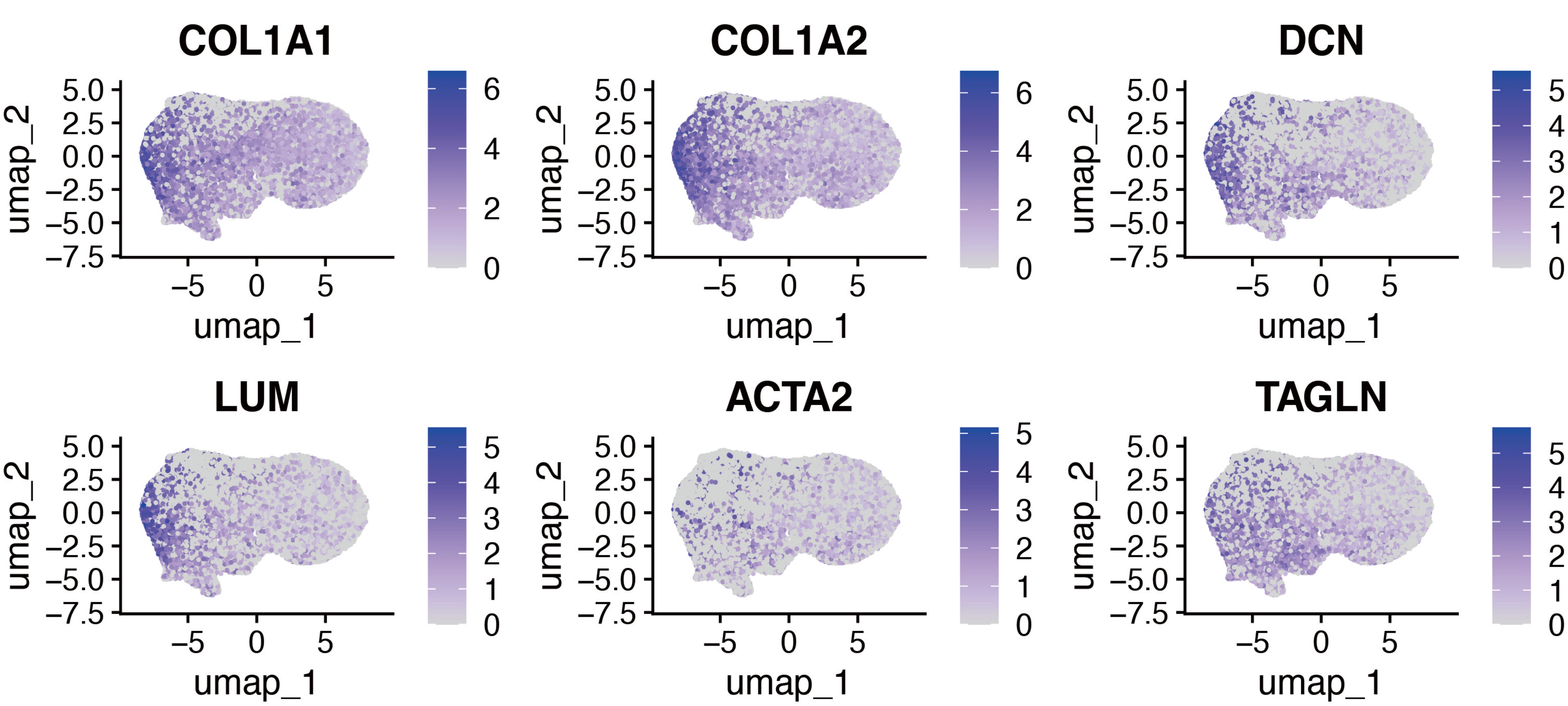
COL1A1
COL1A2
DCN
LUM
ACTA2
TAGLN
umap_1
umap_2

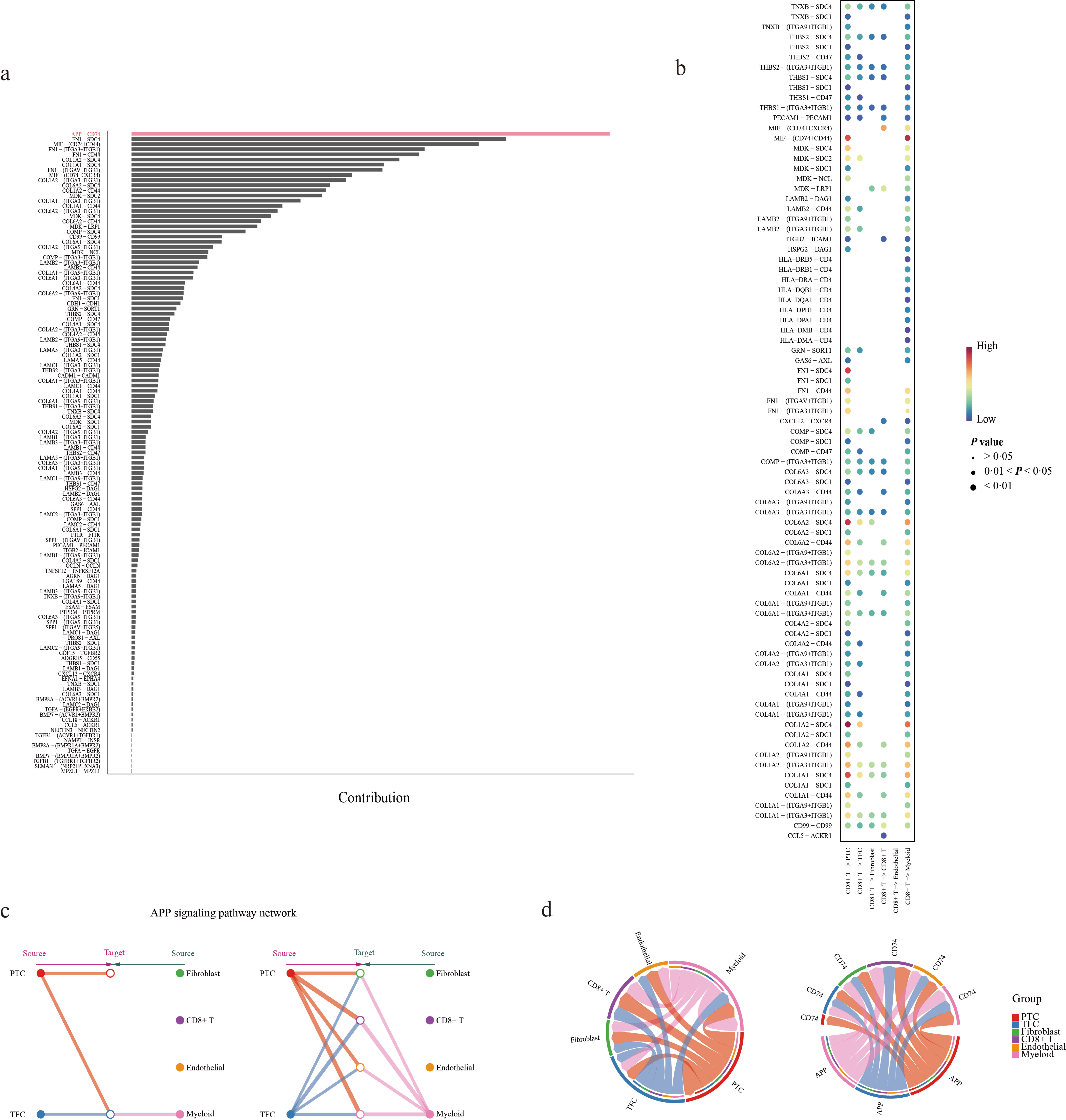

a
APP – CD74
FN1 – SDC4
MIF – (CD74+CD44)
FN1 – (ITGA3+ITGB1)
FN1 – CD44
Contribution
b
TNXB – SDC4
TNXB – SDC1
TNXB – (ITGA9+ITGB1)
THBS2 – SDC4
THBS2 – SDC1
THBS2 – CD47
THBS2 – (ITGA3+ITGB1)
THBS1 – SDC4
THBS1 – SDC1
THBS1 – CD47
THBS1 – (ITGA3+ITGB1)
PECAM1 – PECAM1
MIF – (CD74+CXCR4)
MIF – (CD74+CD44)
MDK – SDC4
MDK – SDC2
MDK – SDC1
MDK – NCL
MDK – LRP1
LAMB2 – DAG1
LAMB2 – CD44
LAMB2 – (ITGA9+ITGB1)
LAMB2 – (ITGA3+ITGB1)
ITGB2 – ICAM1
HSPG2 – DAG1
HLA–DRB5 – CD4
HLA–DRB1 – CD4
HLA–DRA – CD4
HLA–DQB1 – CD4
HLA–DQA1 – CD4
HLA–DPB1 – CD4
HLA–DPA1 – CD4
HLA–DMB – CD4
HLA–DMA – CD4
GRN – SORT1
GAS6 – AXL
FN1 – SDC4
FN1 – SDC1
FN1 – CD44
FN1 – (ITGAV+ITGB1)
FN1 – (ITGA3+ITGB1)
CXCL12 – CXCR4
COMP – SDC4
COMP – SDC1
COMP – CD47
COMP – (ITGA3+ITGB1)
COL6A3 – SDC4
COL6A3 – SDC1
COL6A3 – CD44
COL6A3 – (ITGA9+ITGB1)
COL6A3 – (ITGA3+ITGB1)
COL6A2 – SDC4
COL6A2 – SDC1
COL6A2 – CD44
COL6A2 – (ITGA9+ITGB1)
COL6A2 – (ITGA3+ITGB1)
COL6A1 – SDC4
COL6A1 – SDC1
COL6A1 – CD44
COL6A1 – (ITGA9+ITGB1)
COL6A1 – (ITGA3+ITGB1)
COL4A2 – SDC4
COL4A2 – SDC1
COL4A2 – CD44
COL4A2 – (ITGA9+ITGB1)
COL4A2 – (ITGA3+ITGB1)
COL4A1 – SDC4
COL4A1 – SDC1
COL4A1 – CD44
COL4A1 – (ITGA9+ITGB1)
COL4A1 – (ITGA3+ITGB1)
COL1A2 – SDC4
COL1A2 – SDC1
COL1A2 – CD44
COL1A2 – (ITGA9+ITGB1)
COL1A2 – (ITGA3+ITGB1)
COL1A1 – SDC4
COL1A1 – SDC1
COL1A1 – CD44
COL1A1 – (ITGA9+ITGB1)
COL1A1 – (ITGA3+ITGB1)
CD99 – CD99
CCL5 – ACKR1
CD8+ T -> PTC
CD8+ T -> TFC
CD8+ T -> Fibroblast
CD8+ T -> CD8+ T
CD8+ T -> Endothelial
CD8+ T -> Myeloid
High
Low
P value
> 0·05
0·01 < P < 0·05
< 0·01
c
APP signaling pathway network
Source
Target
Source
PTC
TFC
Fibroblast
CD8+ T
Endothelial
Myeloid
d
Endothelial
Myeloid
CD8+ T
Fibroblast
TFC
PTC
CD74
APP
Group
PTC
TFC
Fibroblast
CD8+ T
Endothelial
Myeloid

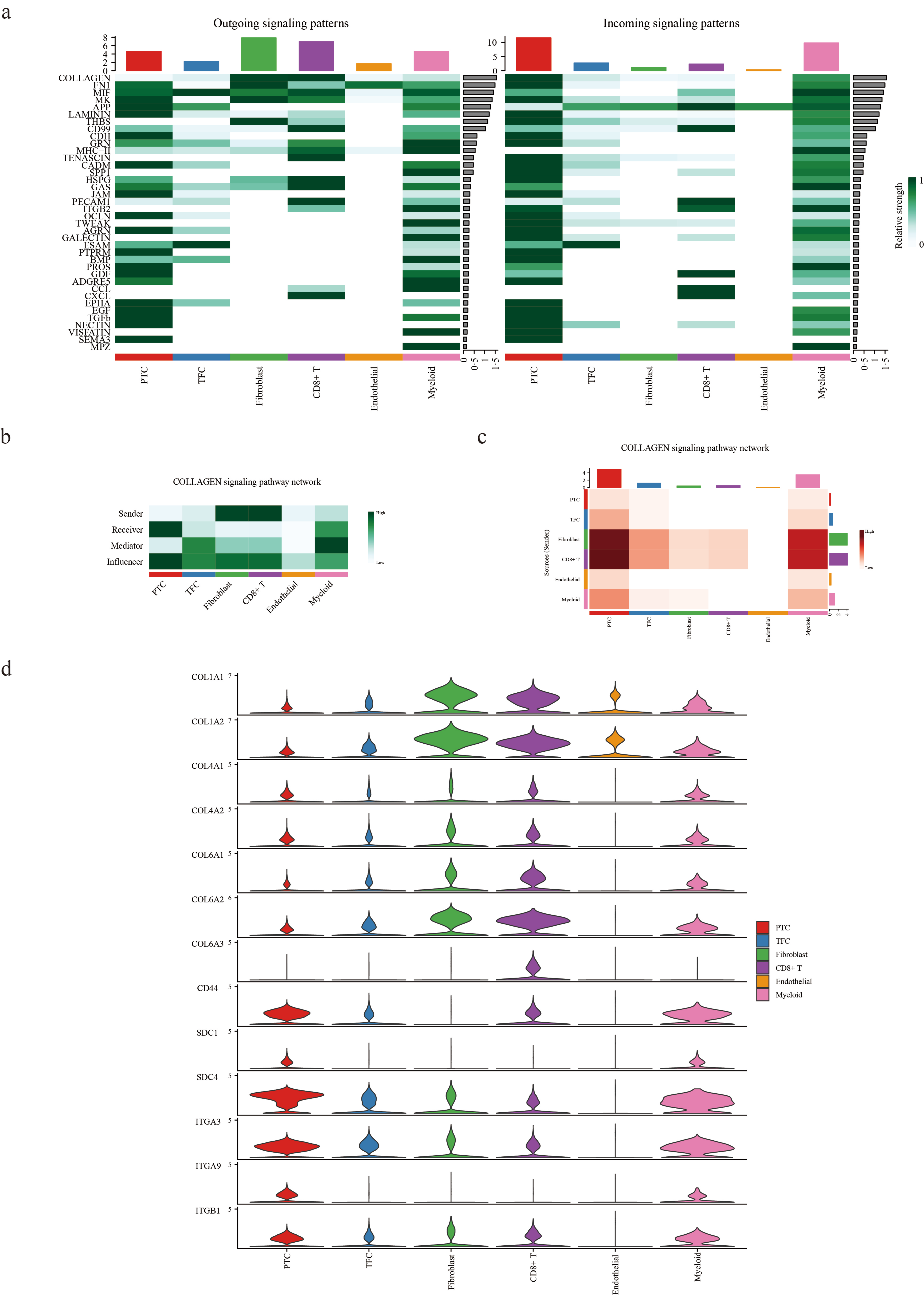
a
Outgoing signaling patterns
Incoming signaling patterns
COLLAGEN
FN1
MIF
MK
APP
LAMININ
THBS
CD99
CDH
GRN
MHC−II
TENASCIN
CADM
SPP1
HSPG
GAS
JAM
PECAM1
ITGB2
OCLN
TWEAK
AGRN
GALECTIN
ESAM
PTPRM
BMP
PROS
GDF
ADGRE5
CCL
CXCL
EPHA
EGF
TGFb
NECTIN
VISFATIN
SEMA3
MPZ
PTC
TFC
Fibroblast
CD8+ T
Endothelial
Myeloid
Relative strength
b
COLLAGEN signaling pathway network
Sender
Receiver
Mediator
Influencer
High
Low
c
COLLAGEN signaling pathway network
Sources (Sender)
d
COL1A1
COL1A2
COL4A1
COL4A2
COL6A1
COL6A2
COL6A3
CD44
SDC1
SDC4
ITGA3
ITGA9
ITGB1

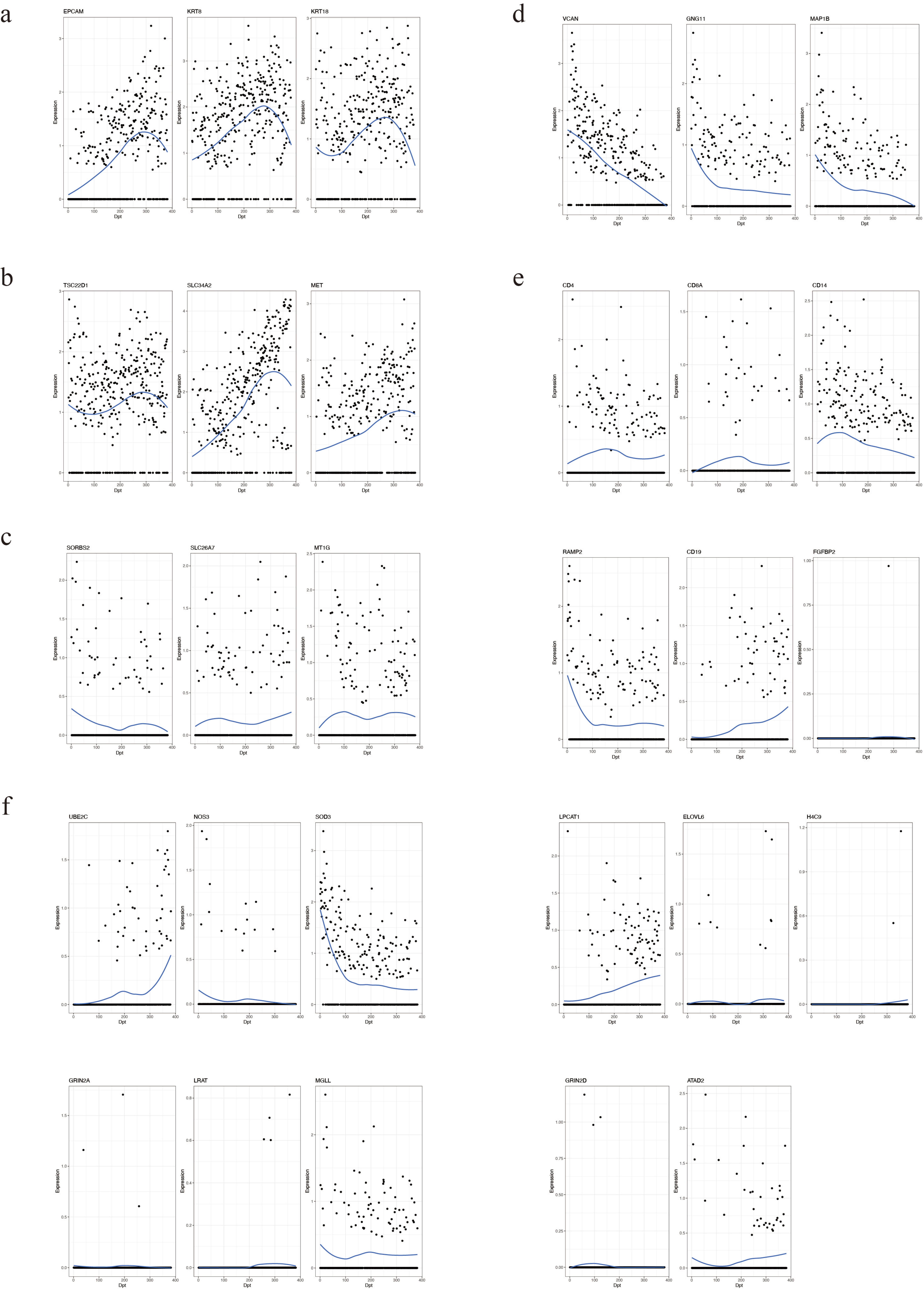
a
EPCAM
KRT8
KRT18
b
TSC22D1
SLC34A2
MET
c
SORBS2
SLC26A7
MT1G
d
VCAN
GNG11
MAP1B
e
CD4
CD8A
CD14
RAMP2
CD19
FGFBP2
f
UBE2C
NOS3
SOD3
LPCAT1
ELOVL6
H4C9
GRIN2A
LRAT
MGLL
GRIN2D
ATAD2
Expression
Dpt

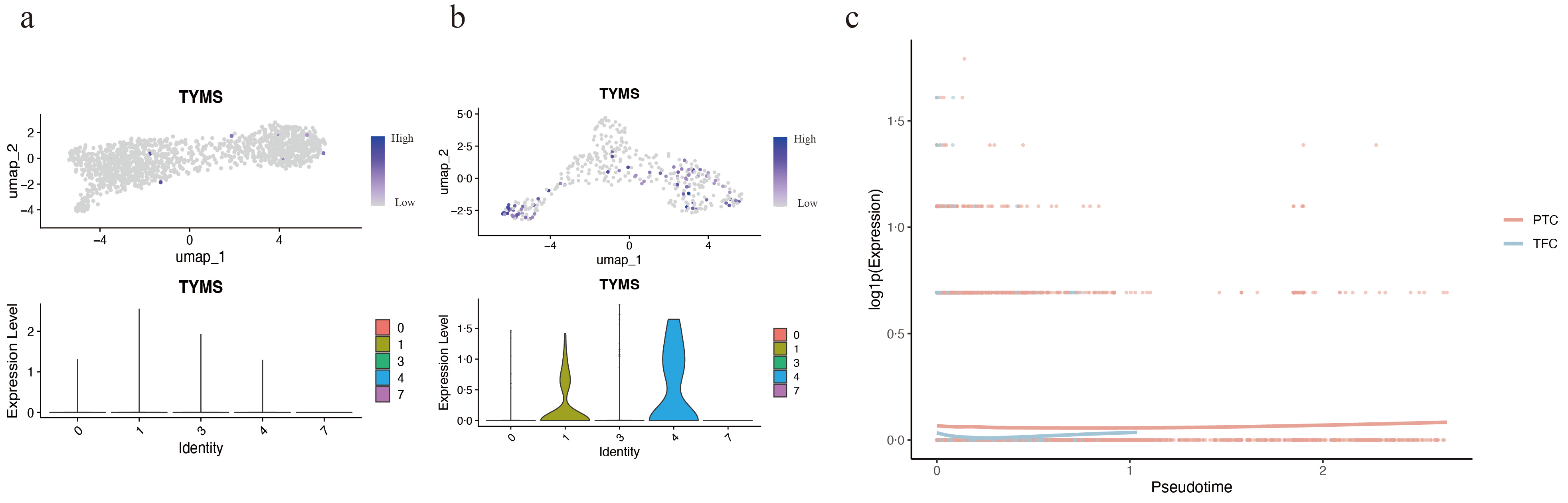
a
TYMS
umap_2
umap_1
High
Low
TYMS
Expression Level
Identity
0
1
3
4
7
b
TYMS
umap_2
umap_1
High
Low
TYMS
Expression Level
Identity
0
1
3
4
7
c
log1p(Expression)
Pseudotime
PTC
TFC

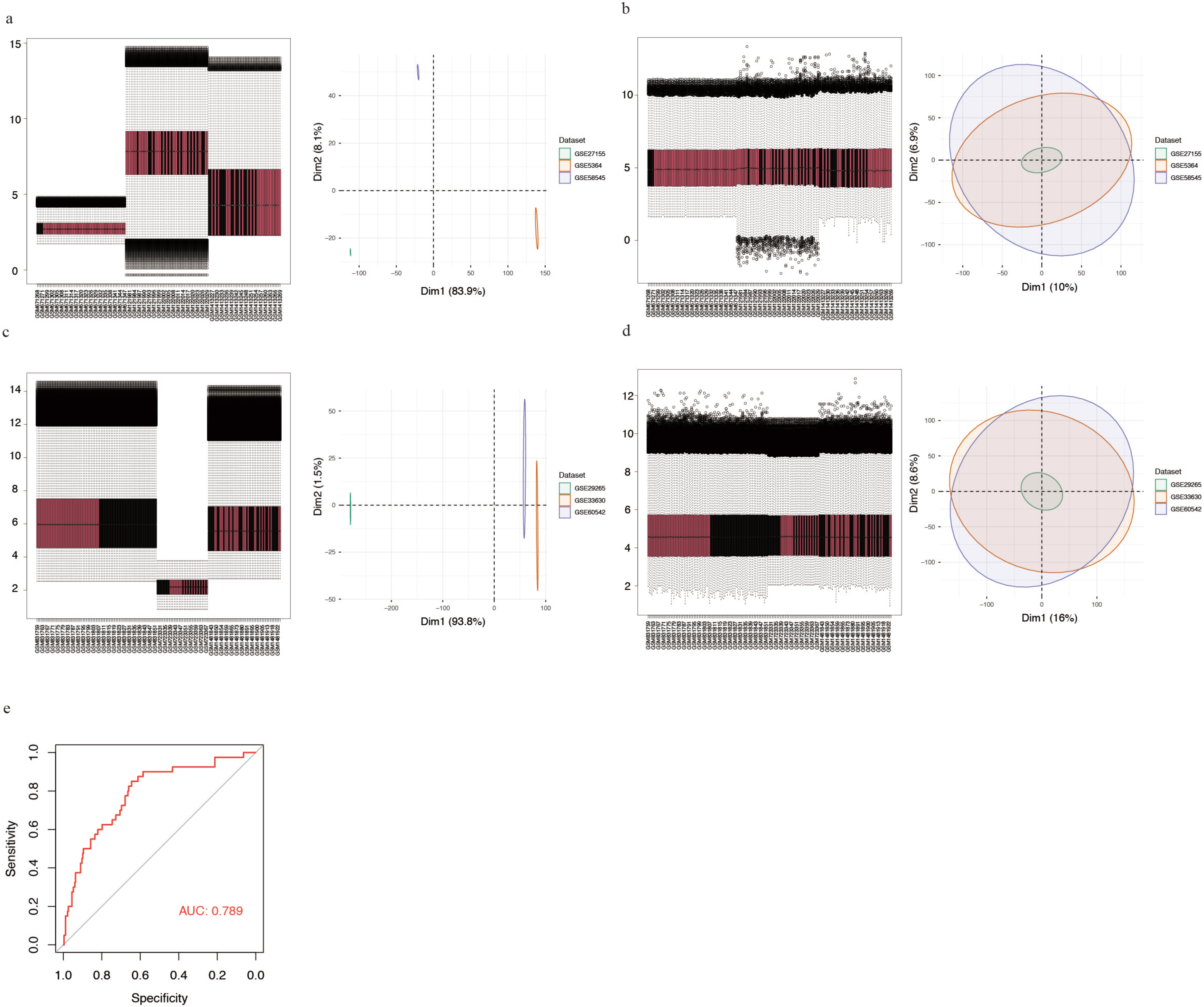
a
Dim2 (8.1%)
Dim1 (83.9%)
Dataset
GSE27155
GSE5364
GSE58545
b
Dim2 (6.9%)
Dim1 (10%)
Dataset
GSE27155
GSE5364
GSE58545
c
Dim2 (1.5%)
Dim1 (93.8%)
Dataset
GSE29265
GSE33630
GSE60542
d
Dim2 (8.6%)
Dim1 (16%)
Dataset
GSE29265
GSE33630
GSE60542
e
Sensitivity
Specificity
AUC: 0.789

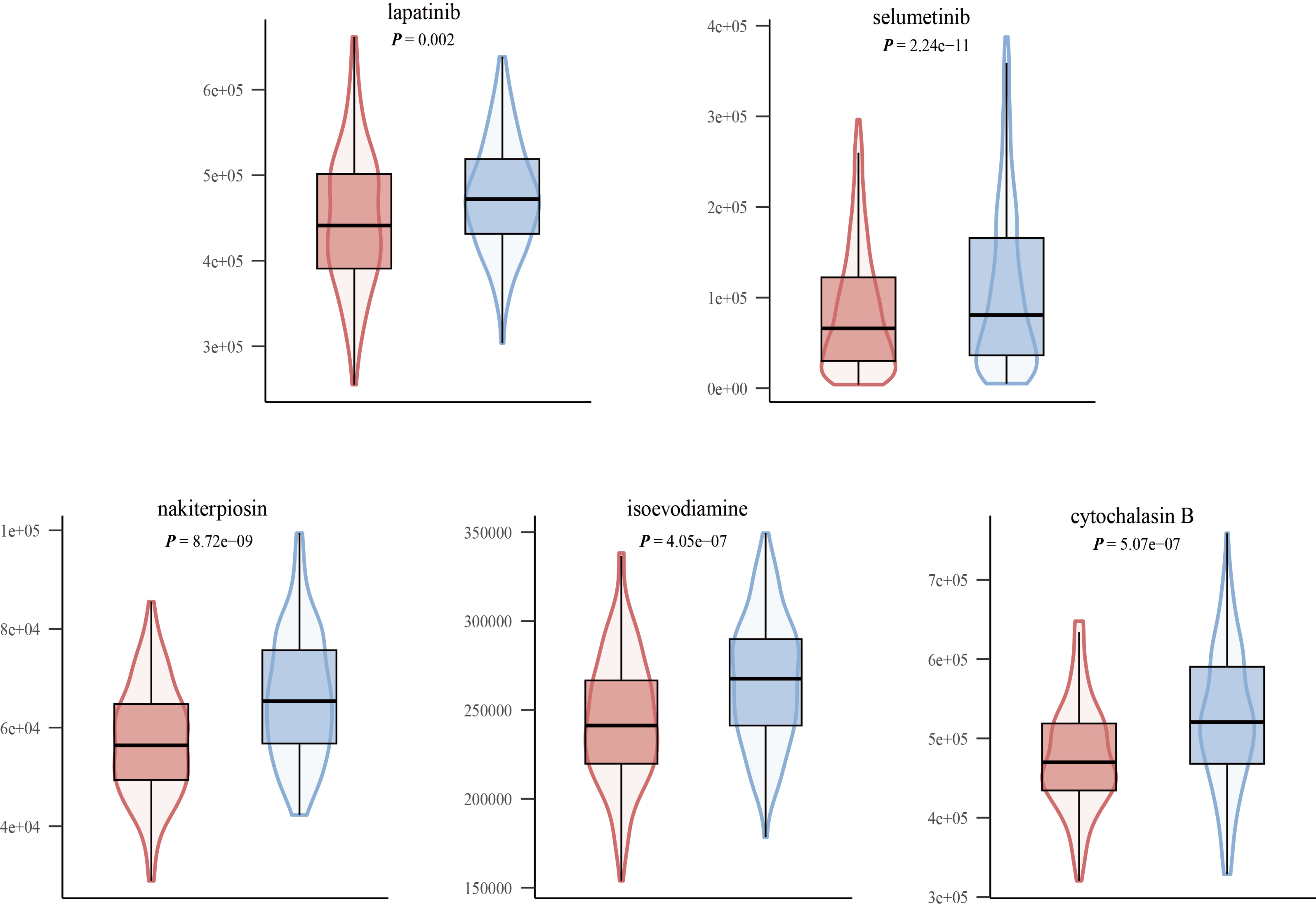

lapatinib
P = 0.002
6e+05
5e+05
4e+05
3e+05
selumetinib
P = 2.24e−11
4e+05
3e+05
2e+05
1e+05
0e+00
nakiterpiosin
P = 8.72e−09
1e+05
8e+04
6e+04
4e+04
isoevodiamine
P = 4.05e−07
350000
300000
250000
200000
150000
cytochalasin B
P = 5.07e−07
7e+05
6e+05
5e+05
4e+05
3e+05
