# Supplementary material for: Integrative profiling of diverse post-translational modifications for prognostic stratification and personalized therapy in papillary thyroid cancer
Source: Front Endocrinol (Lausanne). 2026 Jul 27;17:1875494. doi: 10.3389/fendo.2026.1875494 (PMC13454233; doi:10.3389/fendo.2026.1875494)
Supplement: Supplementary file 3 [file DataSheet3.pdf]

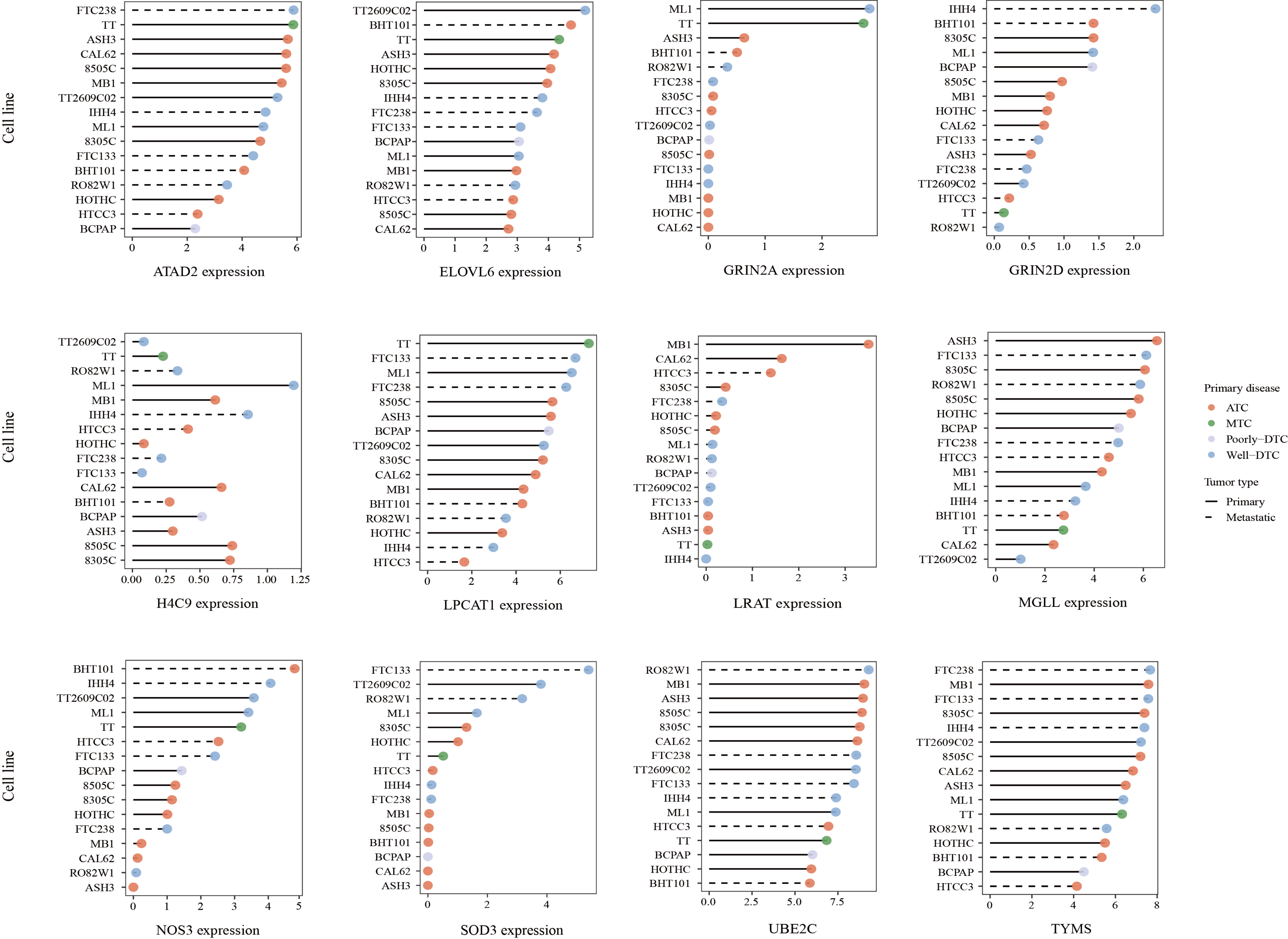
Cell line
ATAD2 expression
ELOVL6 expression
GRIN2A expression
GRIN2D expression
H4C9 expression
LPCAT1 expression
LRAT expression
MGLL expression
NOS3 expression
SOD3 expression
UBE2C
TYMS
Primary disease
ATC
MTC
Poorly−DTC
Well−DTC
Tumor type
Primary
Metastatic

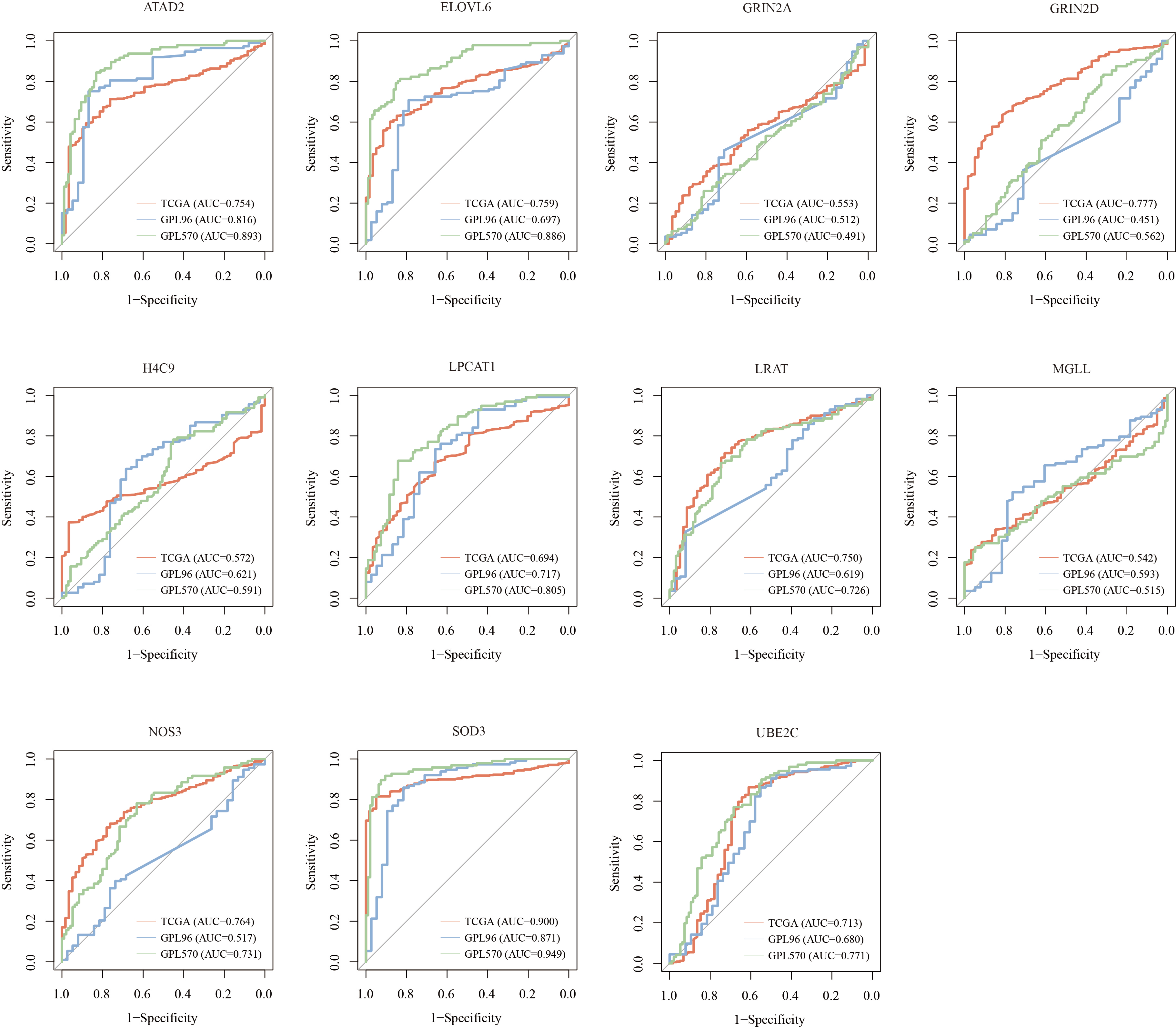
ATAD2
TCGA (AUC=0.754)
GPL96 (AUC=0.816)
GPL570 (AUC=0.893)
ELOVL6
TCGA (AUC=0.759)
GPL96 (AUC=0.697)
GPL570 (AUC=0.886)
GRIN2A
TCGA (AUC=0.553)
GPL96 (AUC=0.512)
GPL570 (AUC=0.491)
GRIN2D
TCGA (AUC=0.777)
GPL96 (AUC=0.451)
GPL570 (AUC=0.562)
H4C9
TCGA (AUC=0.572)
GPL96 (AUC=0.621)
GPL570 (AUC=0.591)
LPCAT1
TCGA (AUC=0.694)
GPL96 (AUC=0.717)
GPL570 (AUC=0.805)
LRAT
TCGA (AUC=0.750)
GPL96 (AUC=0.619)
GPL570 (AUC=0.726)
MGLL
TCGA (AUC=0.542)
GPL96 (AUC=0.593)
GPL570 (AUC=0.515)
NOS3
TCGA (AUC=0.764)
GPL96 (AUC=0.517)
GPL570 (AUC=0.731)
SOD3
TCGA (AUC=0.900)
GPL96 (AUC=0.871)
GPL570 (AUC=0.949)
UBE2C
TCGA (AUC=0.713)
GPL96 (AUC=0.680)
GPL570 (AUC=0.771)
Sensitivity
1−Specificity

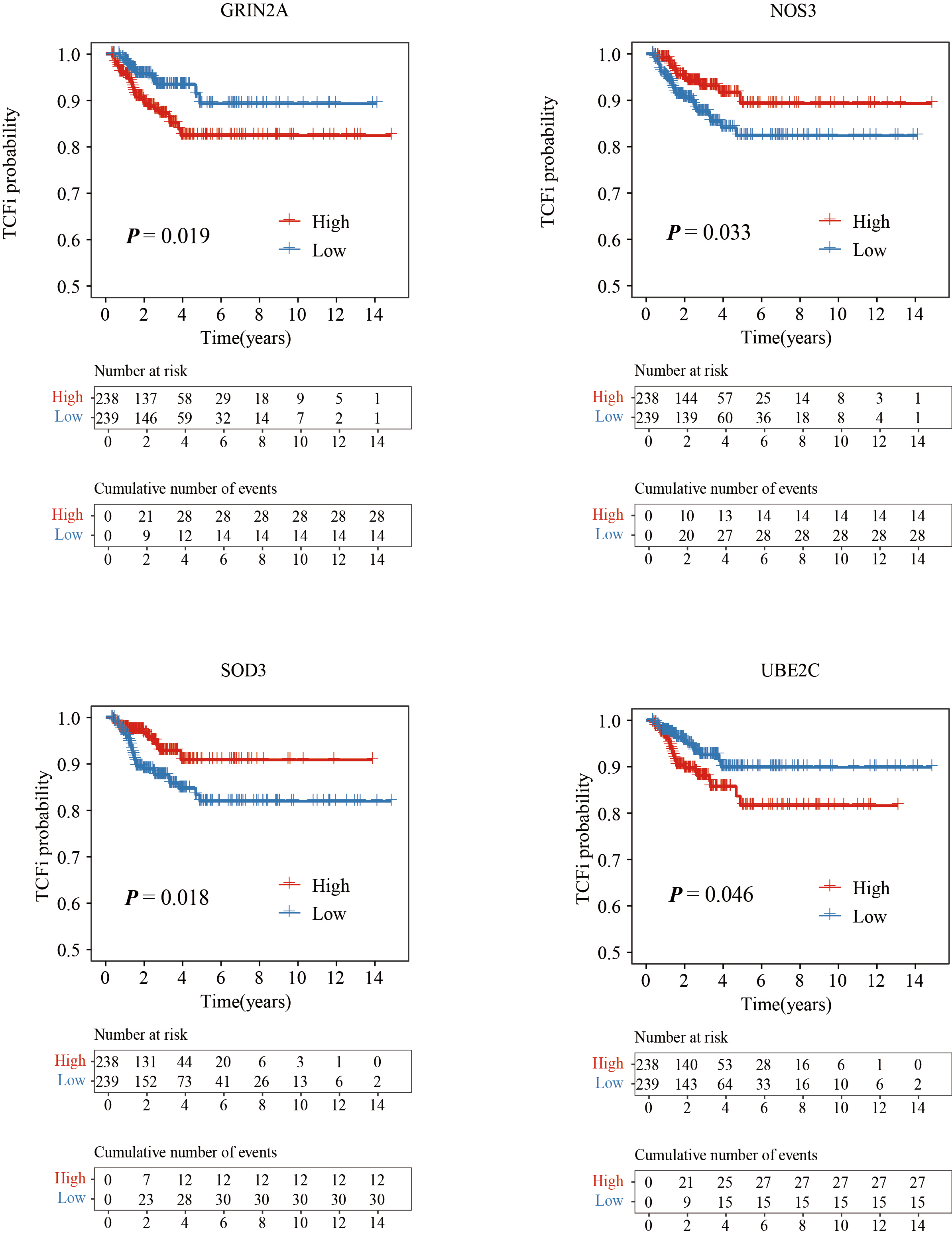
GRIN2A
TCFi probability
1.0
0.9
0.8
0.7
0.6
0.5
P = 0.019
High
Low
0 2 4 6 8 10 12 14
Time(years)
Number at risk
High 238 137 58 29 18 9 5 1
Low 239 146 59 32 14 7 2 1
0 2 4 6 8 10 12 14
Cumulative number of events
High 0 21 28 28 28 28 28 28
Low 0 9 12 14 14 14 14 14
0 2 4 6 8 10 12 14
NOS3
TCFi probability
1.0
0.9
0.8
0.7
0.6
0.5
P = 0.033
High
Low
0 2 4 6 8 10 12 14
Time(years)
Number at risk
High 238 144 57 25 14 8 3 1
Low 239 139 60 36 18 8 4 1
0 2 4 6 8 10 12 14
Cumulative number of events
High 0 10 13 14 14 14 14 14
Low 0 20 27 28 28 28 28 28
0 2 4 6 8 10 12 14
SOD3
TCFi probability
1.0
0.9
0.8
0.7
0.6
0.5
P = 0.018
High
Low
0 2 4 6 8 10 12 14
Time(years)
Number at risk
High 238 131 44 20 6 3 1 0
Low 239 152 73 41 26 13 6 2
0 2 4 6 8 10 12 14
Cumulative number of events
High 0 7 12 12 12 12 12 12
Low 0 23 28 30 30 30 30 30
0 2 4 6 8 10 12 14
UBE2C
TCFi probability
1.0
0.9
0.8
0.7
0.6
0.5
P = 0.046
High
Low
0 2 4 6 8 10 12 14
Time(years)
Number at risk
High 238 140 53 28 16 6 1 0
Low 239 143 64 33 16 10 6 2
0 2 4 6 8 10 12 14
Cumulative number of events
High 0 21 25 27 27 27 27 27
Low 0 9 15 15 15 15 15 15
0 2 4 6 8 10 12 14
