# Supplementary material for: Integrative profiling of diverse post-translational modifications for prognostic stratification and personalized therapy in papillary thyroid cancer
Source: Front Endocrinol (Lausanne). 2026 Jul 27;17:1875494. doi: 10.3389/fendo.2026.1875494 (PMC13454233; doi:10.3389/fendo.2026.1875494)
Supplement: Supplementary file 4 [file SupplementaryFile1.docx]

Supplementary Fig.1 Heatmap of the top50 PTM-DEGs between PTC and normal tissues.

Supplementary Fig.2 Line plot shows TMB Score of PTM related genes in the TCGA cohort.

Supplementary Fig.3 Bubble plot of the relationship among model genes.*P<0·05.

Supplementary Fig.4 PTMI distribution in each set.(a)PTMI distribution and prognostic status of each patient in training set and testing set.(b)PCA plot and t-SNE plot show the separation of PTMI subgroups in training set.(c)PCA plot and t-SNE plot show the separation of PTMI subgroups in testing set.

Supplementary Fig.5 Construction of SHAP model.(a)Partial dependence plot of PTMI subgroups in multi-models.(b)Partial dependence plot of PTMI subgroups in GBM model.(c)Individual conditional expectation on GBM model in PTC patients.
(d)Learning curve of GBM model in training set.(e)TCFi ROC curve of SHAP value in the training set.(f)Interaction effect diagram of M Stage in the model.(g-h)The force plot of patients with barcode TCGA-4C-A93U(TCFi=0·353) and TCGA-BJ-A0Z0(TCFi=1·148).(i-j)The feature importance of patients with barcode TCGA-4C-A93U(TCFi=0·353) and TCGA-BJ-A0Z0 (TCFi=1·148).

Supplementary Fig.6 Quality control of single-cell RNA sequencing (scRNA) data from 9 single-cell datasets.(a)The gene counts per cell (nFeature_RNA), number of unique molecular identifiers (UMIs) per cell (nCount_RNA), and percentage of mitochondrial genes per cell (percent. mt) of the scRNA-seq data.(b)The variance plot showed 22428 genes in all cells, red dots represent the top 2000 highly variable genes.(c)UMAP plots for the identification of single cells in 9 datasets.(d)Heatmap of thyroid function gene expression in PTC and TFC cells.(e)Pseudotime across 6 cell types.(f)Heatmap of model genes expression in 6 cell types.****P < 0·0001.

Supplementary Fig.7 GO enrichment analyses based on the top30 highly variable genes.

Supplementary Fig.8 Feature plots present the typical PTC marker genes expression.

Supplementary Fig.9 Feature plots present the typical marker gene expression.(a-c)The distribution and expressions of TFC,myeloid ,T-cells marker genes across different cell types.

Supplementary Fig.10 Feature plots present the typical marker gene expression.(a-b)The distribution and expressions of mesenchymal and fibroblast marker genes across different cell types.

Supplementary Fig.11 Cell–cell communication among 6 cell types.(a)Contribution of each L−R pair.(b)Dot plot showing the APP signaling pathway receptor–ligand interactions in subcellular clusters.(c)Hierarchy plot of APP signaling pathway network.(d)Chord diagram showing the cell–cell interactions of APP signaling pathway in 6 cell types.

Supplementary Fig.12 PTC progression is associated with COLLAGEN signaling pathway.(a)Summary of signaling patterns among 6 cell types.(b)Heatmap shows the relative importance of each group based on the computed four network centrality measures of COLLAGEN signaling network.(c)Heatmap of interactions of COLLAGEN signaling network among 6 cell types.(d)Violin plot shows the distribution and expression of COLLAGEN signaling genes.

Supplementary Fig.13 Dynamics progression of typical marker genes along pseudotime in PTC cells.(a-e)Scatter plots show the expression of epithelial,PTC,TFC,mesenchymal and immune marker genes along inferred trajectory.(f)The dynamics progression of model genes in PTC cells.

Supplementary Fig.14 The expression profiles of TYMS in TFC and PTC cells.(a-b)Feature plots of TYMS in TFC and PTC cells.(c)Line plot of TYMS expression in TFC vs PTC along Pseudotime.

Supplementary Fig.15 Removal of cross-dataset batch effects within each platform. Each panel shows sample-wise expression boxplots (left) and PCA colored by source dataset (right). (a, b)GPL96 (GSE27155, GSE5364, GSE58545), before (a) and after (b) correction; (c, d) GPL570 (GSE33630, GSE29265, GSE60542), before (c) and after (d) correction with removeBatchEffect. (e) The ROC curve of the PTMI to predict the responsiveness of PTC-RAI patients to RAI treatments in entire cohort.

Supplementary Fig.16 The differences of drug IC50 between PTMI subgroups.

Supplementary Fig.17 Lollipop plots illustrating the expression levels of model genes across a panel of thyroid cancer cell lines from CCLE database.

Supplementary Fig.18 ROC curves show the effects of model genes on PTC diagnosis.

Supplementary Fig.19 TCFi in the low- and high- model gene groups.
