# Supplementary material for: Integrative profiling of diverse post-translational modifications for prognostic stratification and personalized therapy in papillary thyroid cancer
Source: Front Endocrinol (Lausanne). 2026 Jul 27;17:1875494. doi: 10.3389/fendo.2026.1875494 (PMC13454233; doi:10.3389/fendo.2026.1875494)
Supplement: Supplementary file 5 [file Table2.docx]

|  | **No RAI (N=169)** | **RAI (N=308)** | **P-value** |
| --- | --- | --- | --- |
| **Age** |  |  |  |
| <55 | 118 (69.8%) | 206 (66.9%) | 0.579 |
| >=55 | 51 (30.2%) | 102 (33.1%) |  |
| **Gender** |  |  |  |
| Female | 136 (80.5%) | 209 (67.9%) | 0.00453 |
| Male | 33 (19.5%) | 99 (32.1%) |  |
| **T category** |  |  |  |
| T1 | 78 (46.2%) | 58 (18.8%) | <0.001 |
| T2 | 53 (31.4%) | 104 (33.8%) |  |
| T3 | 36 (21.3%) | 128 (41.6%) |  |
| T4 | 2 (1.2%) | 18 (5.8%) |  |
| **N category** |  |  |  |
| N0 | 128 (75.7%) | 138 (44.8%) | <0.001 |
| N1 | 41 (24.3%) | 170 (55.2%) |  |
| **M category** |  |  |  |
| M0 | 169 (100%) | 299 (97.1%) | 0.0301 |
| M1 | 0 (0%) | 9 (2.9%) |  |
| **TNM stage** |  |  |  |
| Stage I | 151 (89.3%) | 230 (74.7%) | <0.001 |
| Stage II | 16 (9.5%) | 61 (19.8%) |  |
| Stage III~IV | 2 (1.2%) | 17 (5.5%) |  |
| **TCFi event** |  |  |  |
| No | 164 (97.0%) | 271 (88.0%) | 0.00153 |
| Yes | 5 (3.0%) | 37 (12.0%) |  |
| **Follow-up (days)** |  |  |  |
| Mean (SD) | 1110 (988) | 1210 (940) | 0.12 |
| Median [Min, Max] | 931 [125, 5150] | 917 [122, 5420] |  |
